# Phenoxazine-Type Pigments from *Pycnoporus sanguineus* as ZnTiO_3_/TiO_2_ Sensitizers for DSSCs

**DOI:** 10.3390/molecules31183310

**Published:** 2026-09-18

**Authors:** Ximena Jaramillo-Fierro, Elías Aguilar, John Ramón, Darío Cruz

**Affiliations:** 1 Departamento de Química y Producción, Facultad de Ciencias Exactas y Naturales, Universidad Técnica Particular de Loja, San Cayetano Alto, Loja 1101608, Ecuador; 2 Carrera de Ingeniería Química, Facultad de Ciencias Exactas y Naturales, Universidad Técnica Particular de Loja, San Cayetano Alto, Loja 1101608, Ecuador; 3 Microbial Systems Ecology and Evolution Research Group, Department of Biological Sciences, Biology School, Universidad Técnica Particular de Loja, San Cayetano Alto s/n C.P., Loja 1101608, Ecuador; djcruz@utpl.edu.ec

**Keywords:** DSSC, fungal pigments, HPLC-DAD, Cinnabarin, Cinnabarinic acid, ZnTiO_3_/TiO_2_, DFT

## Abstract

Dye-sensitized solar cells (DSSCs) using natural pigments offer a low-cost, non-toxic alternative to ruthenium dyes, but fungal sensitizers remain underexplored relative to plant-derived pigments. This study reports, for the first time, the phenoxazine pigments cinnabarin and cinnabaric acid from *Pycnoporus sanguineus* extract (PSE) as DSSC sensitizers, paired with a ZnTiO_3_/TiO_2_ heterojunction photoanode designed to suppress interfacial recombination. The extract was characterized by HPLC-DAD and FTIR, and the semiconductors ZnTiO_3_/TiO_2_ (ZTO) and TiO_2_ (TO) by XRD and SEM. Four device configurations (TO, PSE/TO, ZTO/TO, PSE/ZTO/TO) were evaluated photovoltaically and compared statistically (ANOVA, Tukey/Duncan), while DFT calculations (Gaussian, VASP) modeled dye electronic structure, band-edge alignment, and surface adsorption. Efficiency increased significantly across all configurations, from 0.06% (TO) to 0.38% (PSE/ZTO/TO). Band-alignment calculations revealed a thermodynamically favorable injection pathway from the dyes into ZnTiO_3_, with TiO_2_ acting as a unidirectional barrier against backtransfer, consistent with the dye-independent increase in open-circuit voltage observed experimentally. Adsorption modeling revealed a shared vertical anchoring geometry with differentiated surface selectivity between the two dyes. These results establish *P. sanguineus* phenoxazines as a competitive, mechanistically characterized addition to the limited catalog of fungal DSSC sensitizers and support ZnTiO_3_/TiO_2_ heterostructure engineering as a key strategy for improving fungal-pigment DSSC performance.

## 1. Introduction

Global efforts to decarbonize energy production have driven decades of continuous research into photovoltaic technologies, from wafer-based crystalline silicon (first generation) to thin-film devices (second generation) and a diverse family of third-generation concepts: organic, perovskite, quantum dot, and dye-sensitized solar cells, which sacrifice some of silicon’s efficiency in exchange for lower cost, lighter weight, and simpler processing [1]. Among these, dye-sensitized solar cells (DSSCs) have been a persistently active area of research since their introduction, due to their low fabrication cost, mechanical flexibility, tolerance to diffuse and low-intensity lighting [2], and comparatively simple assembly relative to silicon or thin-film alternatives [3,4].

A DSSC operates through the coordinated action of four components: a mesoporous semiconductor photoanode (typically TiO_2_), a photosensitizing dye anchored to its surface, an electrolyte containing a redox couple (commonly I^−^/I_3_^−^), and a catalytic counter electrode that regenerates the redox mediator [4,5]. After photon absorption, the dye injects an excited electron into the conduction band of the semiconductor; the resulting oxidized dye is regenerated by the electrolyte, and the reduced redox species diffuses to the counter electrode to close the circuit. Therefore, the overall conversion efficiency depends jointly on light-harvesting capacity, electron injection driving force, interfacial recombination losses, and charge collection efficiency, making each of the four components a key target for independent optimization [4].

Polypyridyl ruthenium complexes (e.g., N719, N3, black dye) remain the gold standard sensitizers, achieving power conversion efficiencies (PCE) close to 12% under standard lighting [3]. Further gains have been achieved through ligand engineering, such as tuning the conjugation of bipyridine ligands in ruthenium complexes [6]. However, their scarcity, high synthesis cost, and environmental toxicity have motivated a substantial and growing research effort toward natural pigments as low-cost, non-toxic, and biodegradable alternatives, extracted directly from readily available biological sources without the need for complex organic synthesis [7,8,9,10,11]. Anthocyanins, betalains, carotenoids, chlorophylls, and flavonoids extracted from fruits, flowers, leaves, and roots have been evaluated as DSSC sensitizers: reported examples include betalain- and anthocyanin-rich extracts [12], barberry pigments and phytolac encompassing anthocyanin, isoquinoline alkaloid, and betalain chemistries with PCE up to 3.04% [13], anthoxanthin-based dyes characterized both experimentally and theoretically [14], fruit extracts of *Eleiodoxa conferta* (Griff.) Burret and *Garcinia atroviridis* Griff. ex T. Anderson [15], and various low-cost plant pigment cells assembled using simple extraction protocols [16]. The literature shows that natural sensitizers usually produce power conversion efficiencies (PCE) much lower than those of synthetic dyes—often below 2–3%—but they retain great practical appeal for low-cost and environmentally friendly photovoltaic applications, provided that their main limitations (narrow and often poorly overlapping absorption bands, weak or unstable anchoring to the semiconductor and susceptibility to photodegradation and thermal degradation) can be mitigated by optimization of extraction, co-sensitization or photoanode engineering [17,18,19,20].

Compared to the extensive literature on plant-derived pigments, microbial sources like fungi remain relatively unexplored despite their favorable attributes, such as controllable and substrate-independent cultivation, diverse and often more photostable chromophore chemistry, and pigment yields independent of seasonal or agricultural limitations [21,22]. A 2021 review cataloging algae, cyanobacteria, bacteria, archaea, and fungi as alternative pigment sources for DSSCs reported photoconversion efficiencies ranging from approximately 0.001% to 4.6% for algal/microalgal sensitizers and from 0.26% to 2.3% specifically for fungal pigments [21]. Direct fungal precedents include extracts from *Cortinarius* species, among which *Cortinarius sanguineus* (Wulfen) Gray achieved the best reported performance (η = 0.64%, FF = 65.9%, J_sc_ = 1.79 mA/cm^2^, V_oc_ = 541 mV; [23]), and melanin pigments extracted from *Sclerotium cepivorum* Berk, *Aspergillus niger* Tiegh and *Albifimbria verrucaria* (Alb. & Schwein.) L. Lombard & Crous, with efficiencies between 0.149% and 0.320% and filling factors up to 0.718 [24]. A narrative review specifically addressing the criteria for viable fungal colorants for DSSCs further underscores both the promise and the persistent limitations—instability, poor semiconductor interaction, and limited cost-effectiveness—of this class of sensitizers [22]. Despite this precedent, the phenoxazine pigments from the commonly known *Pycnoporus sanguineus* (L.) Murrill suggested as a synonym of *Trametes sanguinea* (L.) Imazeki [25]—primarily cinnabarin and cinnabaric acid—have not been previously evaluated, to our knowledge, as DSSC sensitizers, representing an unexplored class of fungal chromophores for this application and the main novelty addressed in the present study.

Regardless of the sensitizer choice, photoanode engineering exerts a strong influence on the overall performance of DSSCs, as charge recombination at the semiconductor/electrolyte interface is a major loss mechanism that limits the open-circuit voltage. This was established in classic studies of band-edge motion and recombination kinetics in nanocrystalline TiO_2_ photoanodes using intensity-modulated photovoltage and photocurrent spectroscopy [26,27] and remains a central design consideration in contemporary photoanode research [28]. Since then, heterojunction and doping strategies have been developed specifically to change the conduction band energetics and suppress this interfacial recombination: Ni-doped TiO_2_/TiO_2_ homojunctions increased PCE from 4.13% to 6.08% through a more negative flat band potential and longer electron lifetime [29]; TiO_2_/g-C_3_N_4_ heterostructure nanosheets combined with Co_9_S_8_ nanotube array counter electrodes achieved an efficiency of 8.07%, with the g-C_3_N_4_ layer acting as an explicit recombination-blocking layer [30]; and reduced graphene oxide-TiO_2_ nanocomposite photoanodes doped with heteroatoms (B or N) improved the PCE from 1.78% (undoped) to 3.94% (doped with N) through a reduced band gap and better charge separation [31], a trend also observed in later work on graphene oxide/TiO_2_ composite photoanodes [32]. In this context, zinc titanate (ZnTiO_3_) has emerged as a promising wide-bandgap perovskite oxide for coupling with TiO_2_. A ZnTiO_3_-reduced graphene oxide nanocomposite photoanode was reported to have more than double the efficiency of unmodified ZnTiO_3_ nanoparticles (1.11% vs. 0.43%) through improved dye adsorption and reduced surface entrapment [33].

At the computational level, density functional theory (DFT) calculations have become standard tools for rationalizing dye-semiconductor interactions and predicting photovoltaic behavior before or during experimental fabrication [34]. Thus, first-principles DFT simulations of polyene-diphenylaniline dyes adsorbed on (ZnTiO_3_)_8_ cluster models have identified the adsorption energy, electron injection driving force, and light-harvesting efficiency as key performance descriptors of ZnTiO_3_-based sensitizers [35]. Unlike these simplified cluster-model studies, the present work explicitly aligns the calculated dye and semiconductor band edges against the real ZnTiO_3_/TiO_2_ heterojunction architecture used experimentally, and computes adsorption energies for both dyes on both oxide surfaces in vertical and horizontal orientations, providing a more direct mechanistic link between DFT predictions and the observed device-level photovoltaic trends.

Taken together, the literature reviewed above establishes two well-founded, convergent design principles: natural (and specifically fungal) sensitizers as viable, low-cost alternatives to synthetic dyes; and oxide-oxide heterojunction photoanodes, especially ZnTiO_3_/TiO_2_, as an effective strategy for suppressing interfacial recombination. Each of these principles has been validated individually in previous work but never combined with the phenoxazine pigments cinnabarin and cinnabaric acid extracted from *Pycnoporus sanguineus*. This raises the central research question addressed in this study: can these phenoxazine pigments function as effective sensitizers for dye-sensitized solar cells (DSSCs) when combined with a ZnTiO_3_/TiO_2_ heterojunction photoanode, and can their performance be mechanistically rationalized at the electronic level? Based on this premise, the present study investigates, for the first time, this fungal extract as a sensitizer for dye-sensitized solar cells (DSSCs) based on a ZnTiO_3_/TiO_2_ heterojunction photoanode. The extract and semiconductors are structurally and spectroscopically characterized, four device configurations (TO, PSE/TO, ZTO/TO, and PSE/ZTO/TO) are statistically evaluated and compared, and their performance is analyzed. DFT calculations of surface adsorption energetics to rationalize observed photovoltaic trends.

## 2. Results

### 2.1. Characterization of the Pycnoporus sanguineus Extract

**Figure 1:**
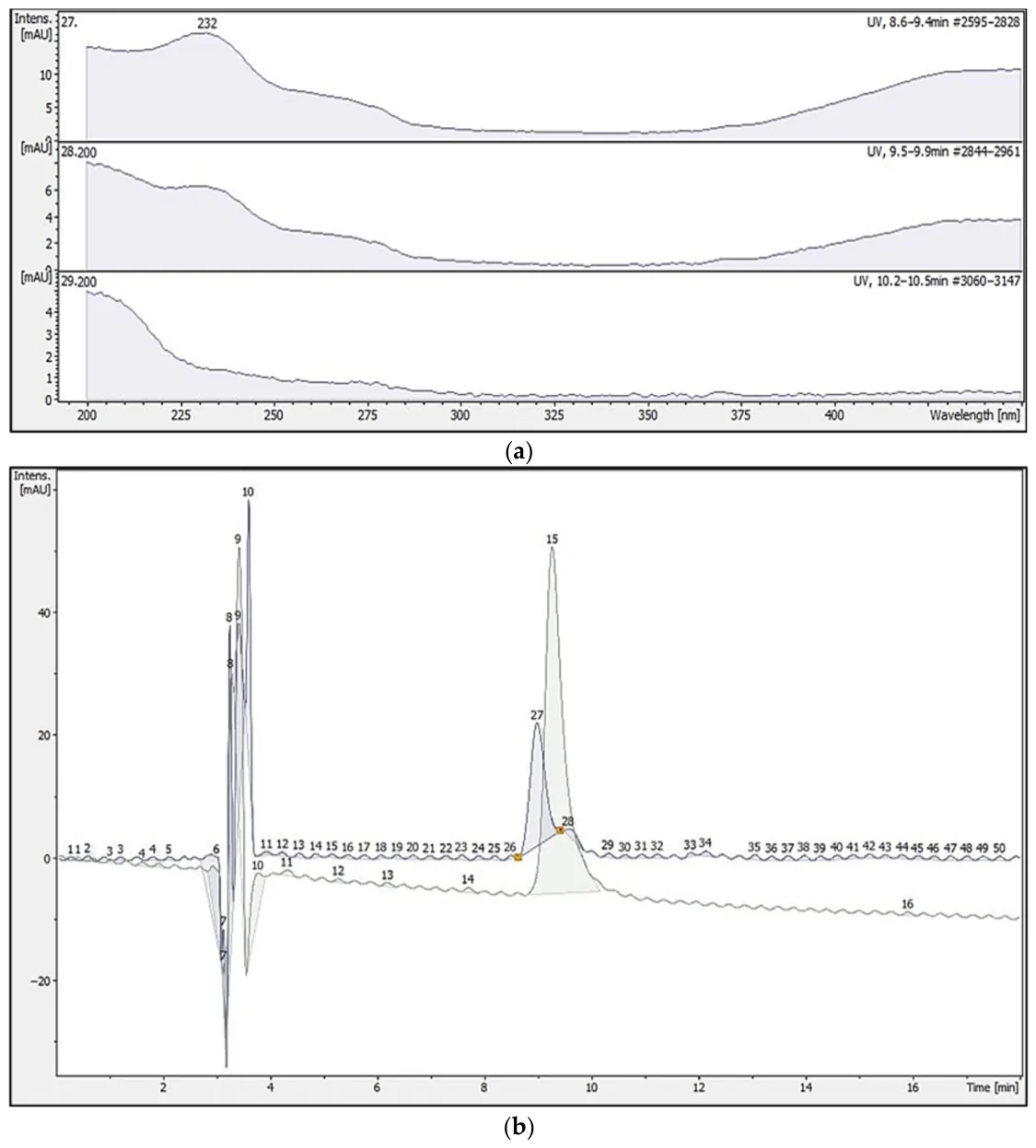
(**a**) UV spectrum and (**b**) UV chromatogram at 430 nm of *Pycnoporus sanguineus* extract.

The UV profile (Figure 1a) of the *Pycnoporus sanguineus* extract, obtained from the HPLC contour plot, showed characteristic UV maxima (λ_max_ 232 and 430 nm) consistent with the presence of a phenoxazone core. Furthermore, the analytical HPLC chromatograms of the extract at 430 nm (Figure 1b) showed peaks between 9.5 and 10 min, suggesting the presence of cinnabaric acid and cinnabarin as the main components of the extract (λ_max_ = ~430 nm). While a cinnabaric acid analytical standard was used for direct comparison, cinnabarin was identified based on its close chromatographic co-elution with cinnabaric acid and its closely matching UV absorption profile (λ_max_~425–448 nm), consistent with reference spectroscopic data reported for both compounds isolated from the related species *Pycnoporus cinnabarinus* [36]. Figure S1 shows the UV spectrum and chromatogram obtained for the corresponding standard.

**Figure 2:**
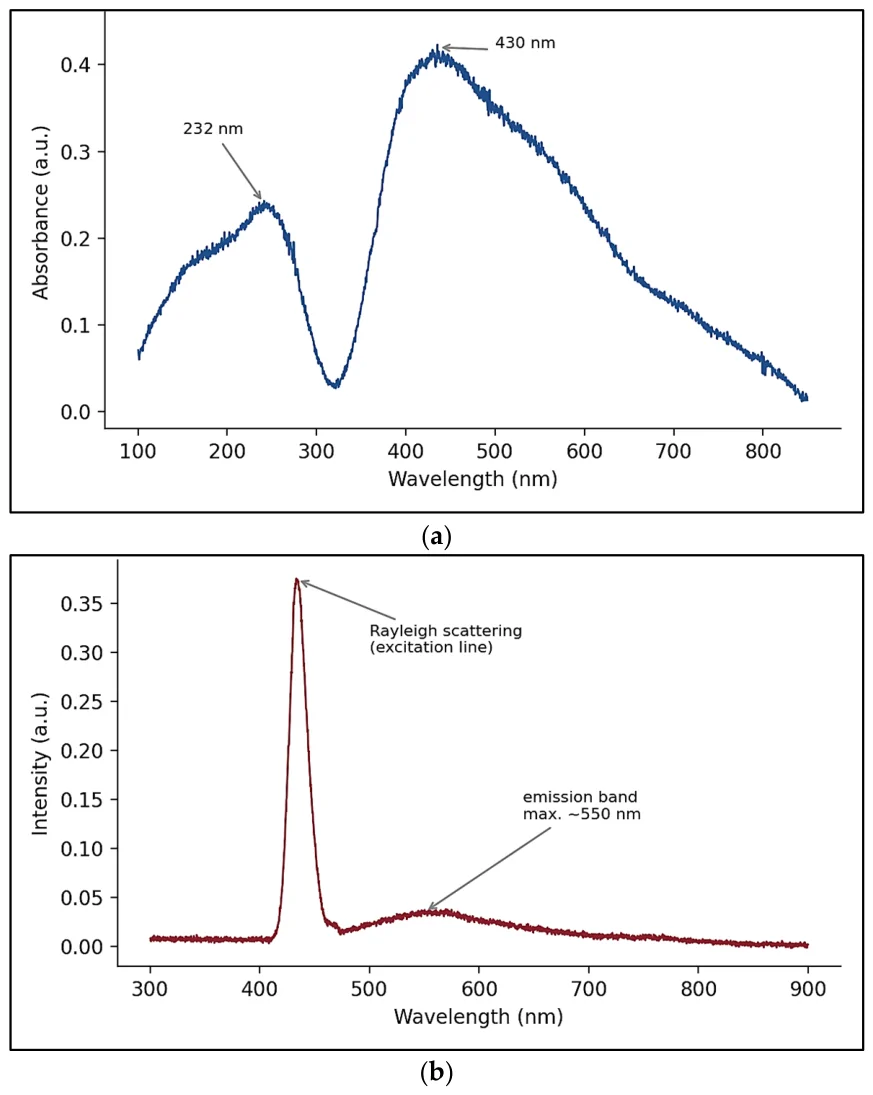
(**a**) UV-Vis absorbance spectrum and (**b**) photoluminescence emission spectrum (λex = 430 nm) of the *Pycnoporus sanguineus* extract.

To directly assess the light-harvesting capacity of the extract, the UV-Vis absorbance spectrum and the photoluminescence (PL) emission spectrum (λ_ex_ = 430 nm) of the *Pycnoporus sanguineus* extract were recorded (Figure 2a and Figure 2b, respectively).

The UV-Vis spectrum confirmed the two absorption maxima already identified from the HPLC-DAD profile (232 and 430 nm; Figure 1a), with the 430 nm band overlapping the visible region of the light source used in the photovoltaic measurements (Figure S2). Under excitation at 430 nm, the PL spectrum showed a broad, low-intensity emission band centered at ~550 nm (Stokes shift of ~120 nm), in addition to the Rayleigh scattering peak from the excitation light. This weak fluorescence could be due to efficient non-radiative decomposition stabilized by intramolecular hydrogen bonds within the chromophore [37].

**Figure 3:**
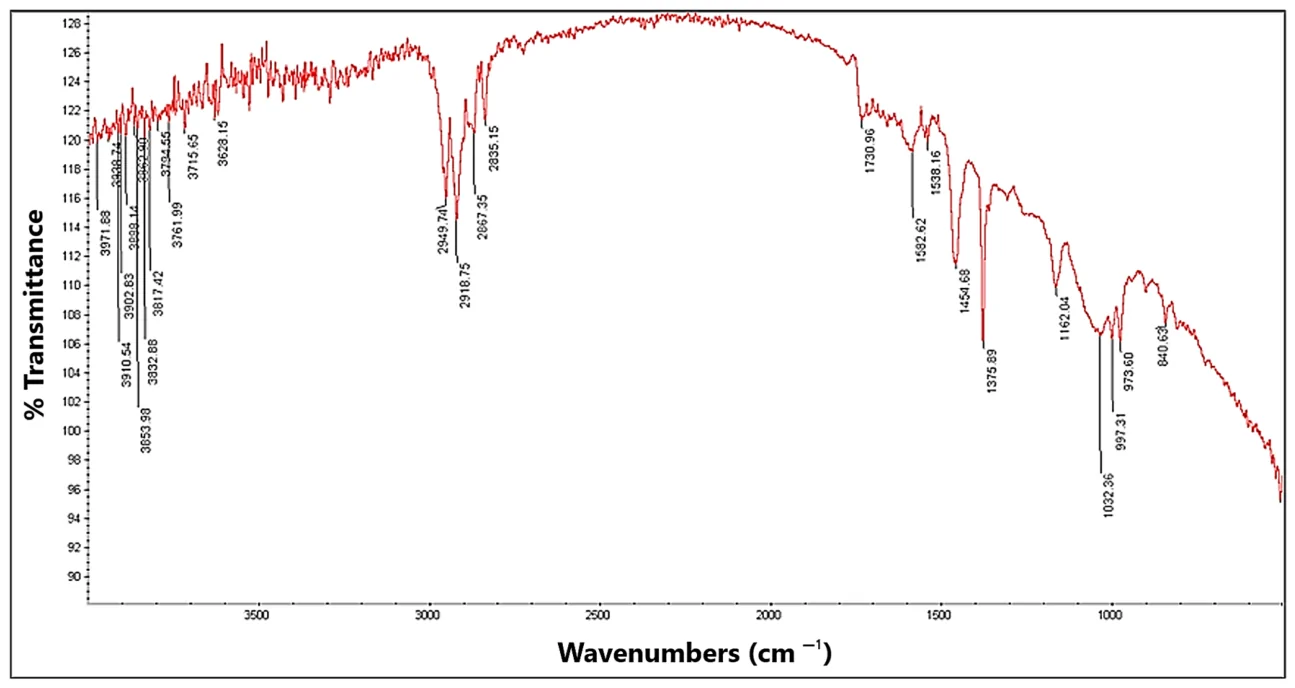
FTIR spectrum of the *Pycnoporus sanguineus* extract.

The FTIR spectrum of the *Pycnoporus sanguineus* extract (Figure 3) showed characteristic hydroxyl and amino group bands in the 3500–3200 cm^−1^ region, as well as aliphatic C–H stretching signals at 2949, 2918, and 2835 cm^−1^. The absorption observed at 1730 cm^−1^ was mainly attributed to C=O stretching of carboxylic groups, consistent with the presence of cinnabaric acid. The bands at 1582 and 1538 cm^−1^ were associated with C=C and C=N vibrations of the conjugated phenoxazine nucleus, while the signals at 1455 and 1376 cm^−1^ were related to ring vibrations, C–N bonds, and carboxylic groups. Finally, the absorptions at 1167 and 1033 cm^−1^ were attributed to C–O and C–N stretching. The correspondence of these bands with those reported for cinnabarin and cinnabaric acid supports the presence of both metabolites in the extract, previously identified as its major components.

### 2.2. Characterization of ZnTiO_3_/TiO_2_ and TiO_2_ Semiconductors

**Figure 4:**
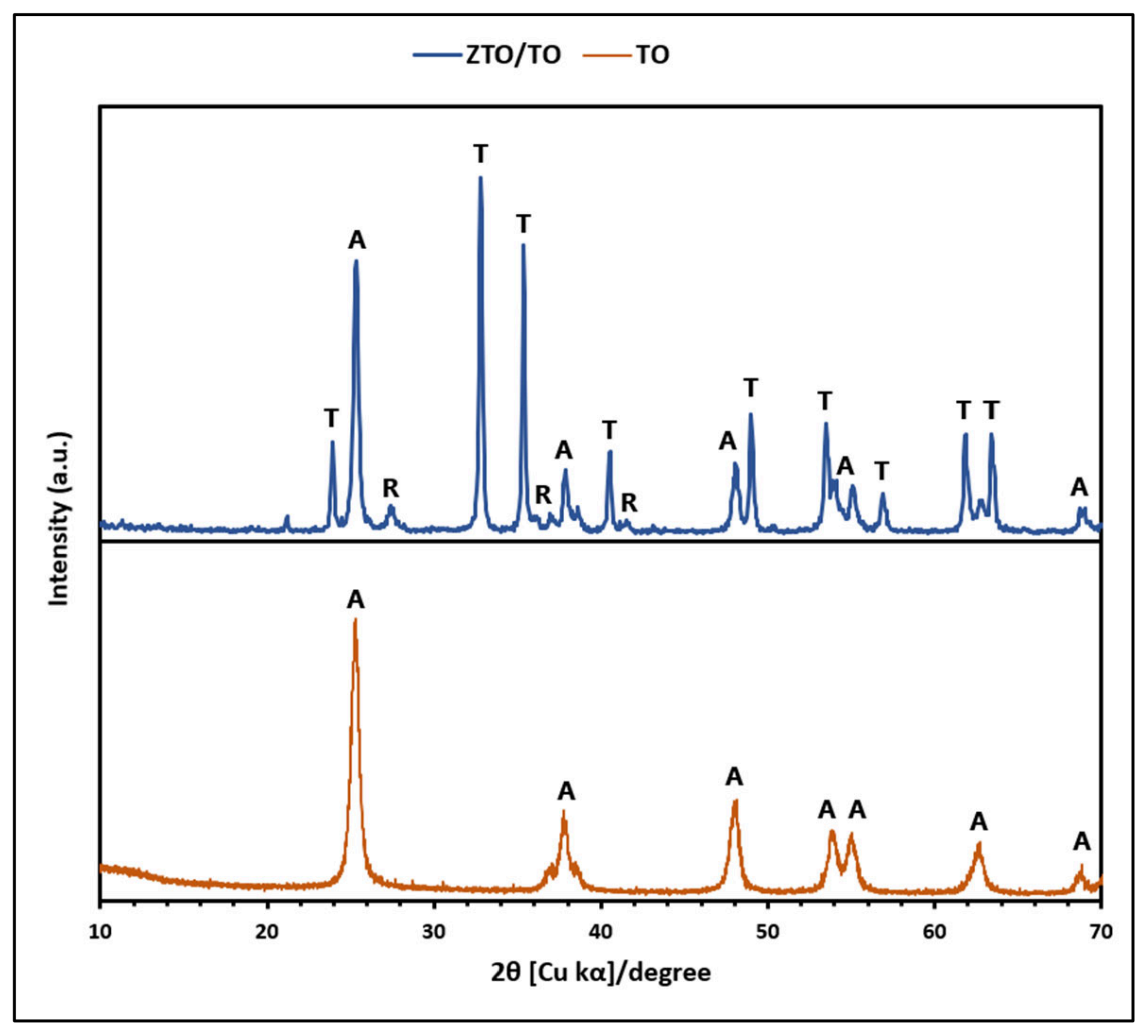
X-ray diffraction (XRD) patterns of ZnTiO_3_/TiO_2_ and TiO_2_ semiconductors. A: Anatase, R: Rutile, T: Zinc titanate.

The diffraction pattern in Figure 4 reveals three distinct crystalline phases present across the ZnTiO_3_/TiO_2_ and TiO_2_ heterostructures. A hexagonal phase (space group R-3, no. 148), consistent with JCPDS card No. 00-015-0591 for ZnTiO_3_, was confirmed with unit cell dimensions of a = b = 5.08 Å and c = 13.93 Å. A second, tetragonal phase matching anatase TiO_2_ (space group I41/amd, no. 141; JCPDS card No. 01-073-1764) was identified with lattice constants a = b = 3.79 Å and c = 9.51 Å. A minor tetragonal rutile phase (space group P42/mnm, no. 136; JCPDS card No. 03-065-0192) was also detected, with corresponding lattice parameters a = b = 4.59 Å and c = 2.96 Å.

Crystal domain sizes for each semiconductor phase were determined by applying the Scherrer equation to the main diffraction peak corresponding to the phase-pure reflections [38].

(1) $$A=\frac{K\lambda }{\beta cos\theta }$$

where K denotes the shape factor (0.89) and λ the X-ray wavelength (0.15406 nm); θ represents the Bragg angle, and β the full width at half maximum (FWHM) of the diffraction peak. Applying this equation yielded average crystal sizes of 27.51 ± 1.27 nm for the ZnTiO_3_ phase and 43.27 ± 1.52 nm for the anatase TiO_2_ phase, the latter averaged across both diffraction patterns in which this phase appears.

**Figure 5:**
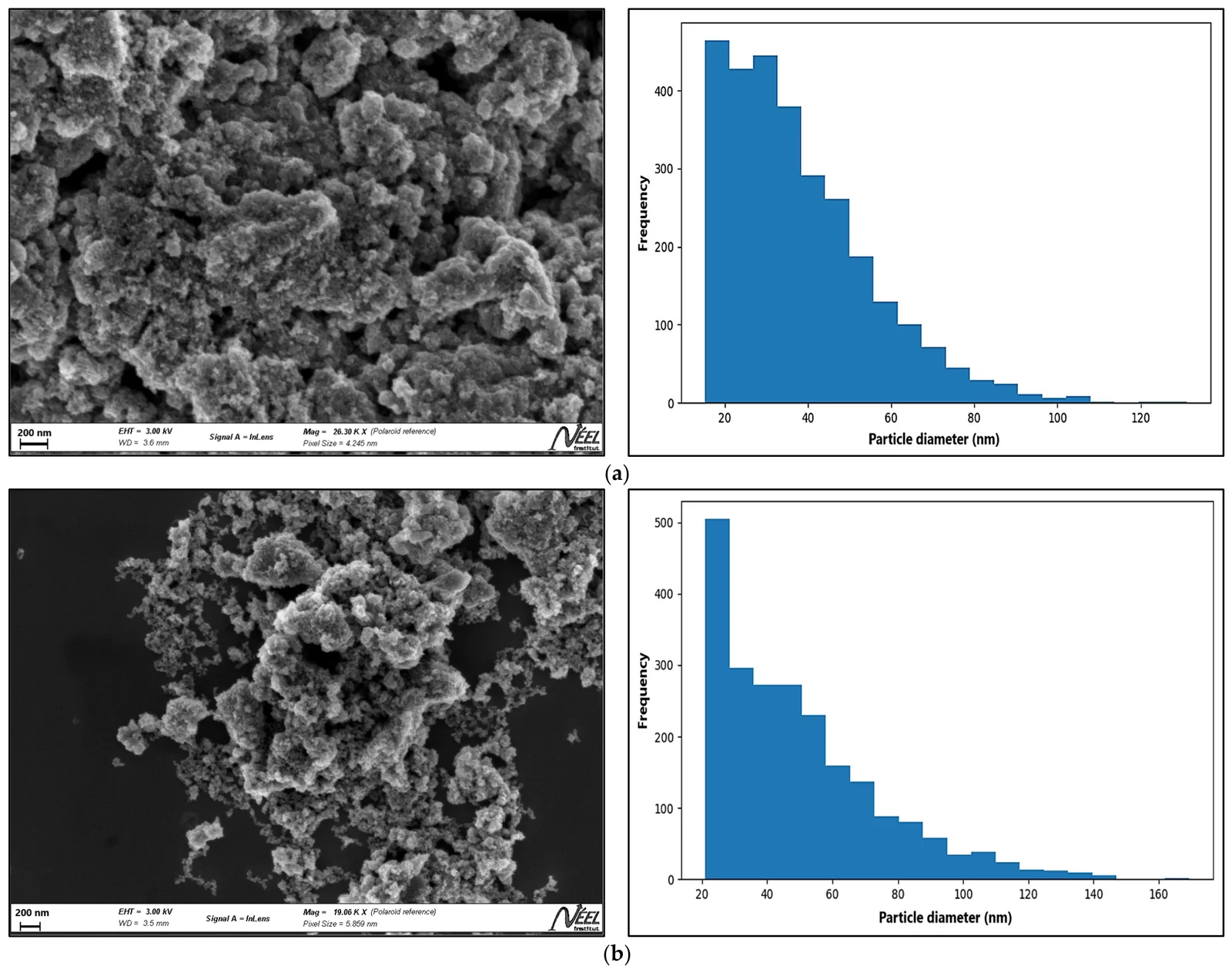
Scanning electron microscopy (SEM) images of (**a**) ZnTiO_3_/TiO_2_ and (**b**) TiO_2_ semiconductors.

On the other hand, scanning electron microscopy (SEM) shows larger particles for TO (~50 nm) than for ZTO/TO (~38 nm) (Figure 5). The sizes were determined using ImageJ version 1.54t, which is a powerful, oft-referenced program for image processing [39]. The image shows that the particles are almost spherical and are highly agglomerated.

Furthermore, the Brunauer–Emmett–Teller (BET) specific surface area analysis results show a higher value for ZTO/TO (87.6 m^2^/g) than for TO (81.4 m^2^/g). These values are somewhat lower, but consistent with those previously reported for nanoparticles synthesized via the same sol–gel route [40,41], which likely reflects a modest increase in particle size between synthesis batches. This larger surface area of ZTO/TO relative to TO is consistent with the improved photovoltaic performance observed for heterojunction devices, along with the electronic advantages of the ZnTiO_3_/TiO_2_ junction described below.

### 2.3. DSSC Performance

**Figure 6:**
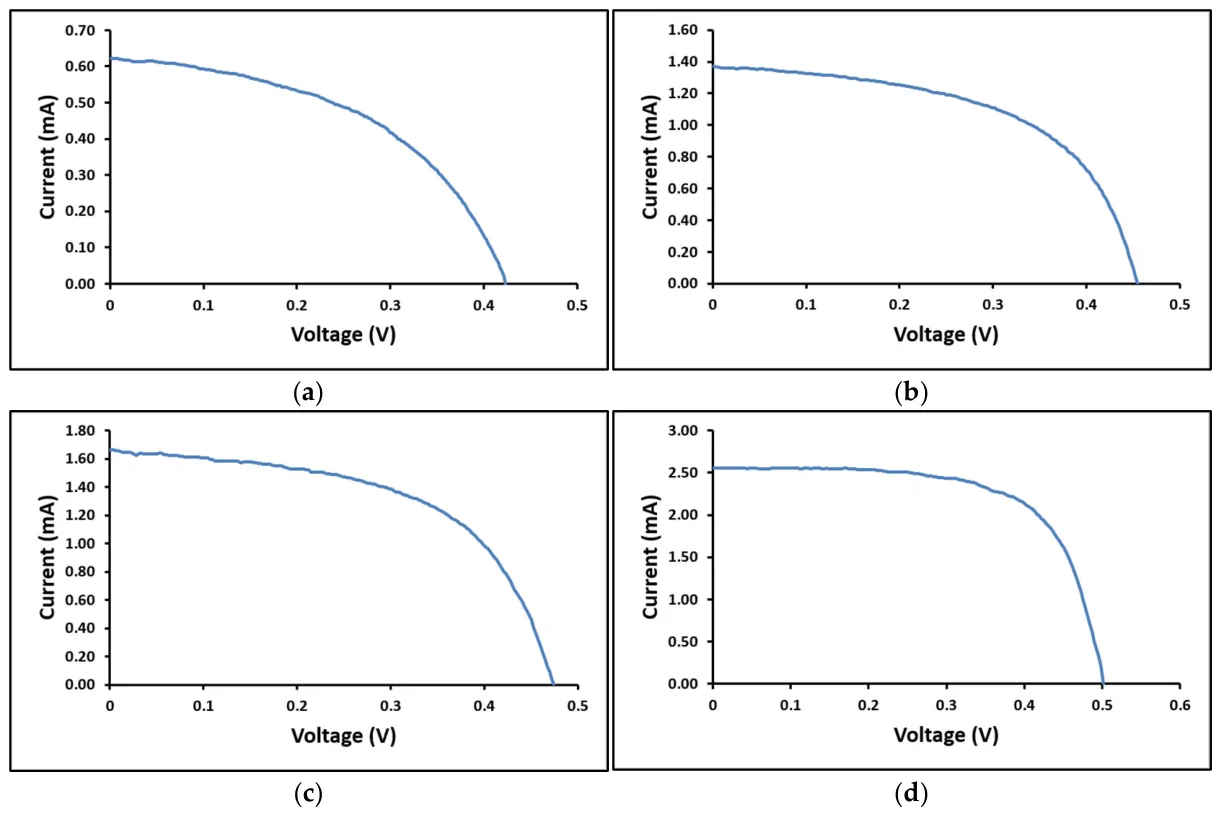
Current (C) vs. voltage (V) curves for cells (**a**) TO, (**b**) PSE/TO, (**c**) ZTO/TO, and (**d**) PSE/ZTO/TO.

Figure 6 shows the current (C) versus voltage (V) curves for each cell sensitized with the *Pycnoporus sanguineus* extract (PSE). These curves show the short-circuit current density (J_sc_) at the Y-intercept and the open-circuit voltage (V_oc_) at the X-intercept.

**Figure 7:**
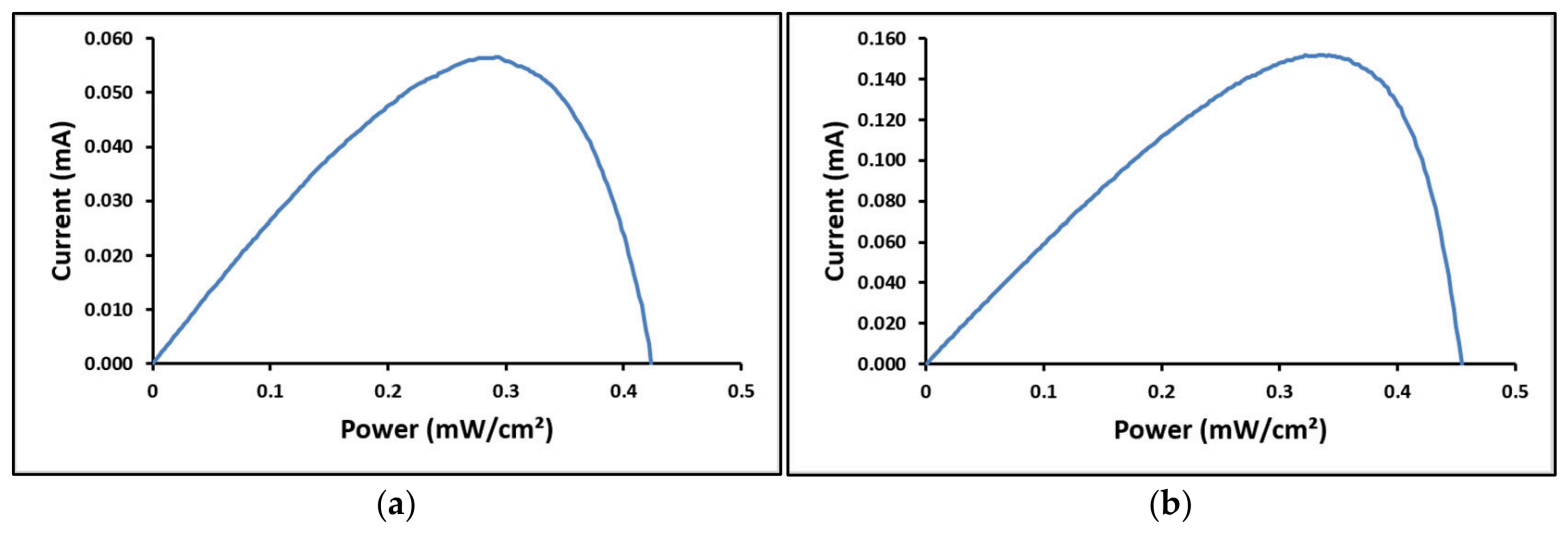

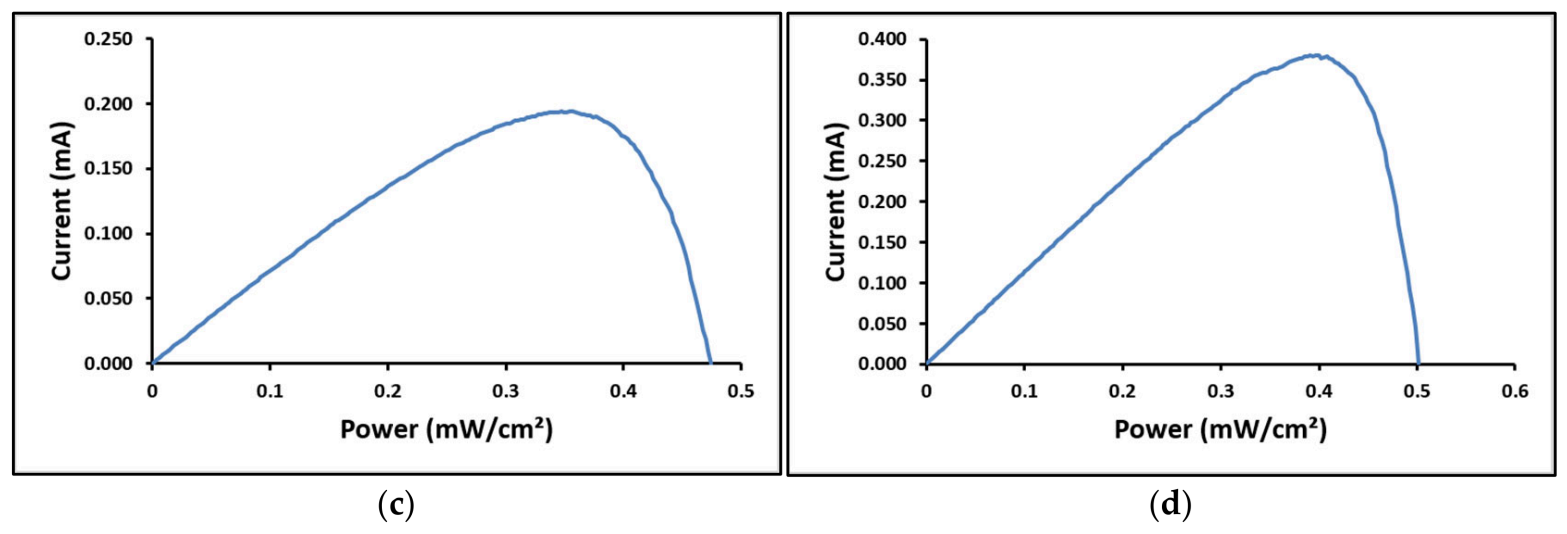
Current (C) vs. power (W) curves for cells (**a**) TO, (**b**) PSE/TO, (**c**) ZTO/TO, and (**d**) PSE/ZTO/TO.

Figure 7 shows the current (C) versus power (W) curves for each cell sensitized with the *Pycnoporus sanguineus* extract (PSE). The curves show a maximum corresponding to the voltage at the maximum power point (V_mp_) and the corresponding current density at the maximum power point (J_mp_).

The characteristic parameters of the DSSCs using the *Pycnoporus sanguineus* extract (PSE) are disclosed in Table 1.

One-way ANOVA was performed for all six photovoltaic descriptors (*n* = 5 per configuration). J_sc_ (F(3, 16) = 798.05, *p* < 0.0001), J_mp_ (F(3, 16) = 1012.34, *p* < 0.0001), and η (F(3, 16) = 1153.44, *p* < 0.0001) were each separated into four statistically distinct groups by Tukey/Duncan HSD post hoc testing (a < b < c < d). V_oc_ (F(3, 16) = 22.67, *p* < 0.0001), FF (F(3, 16) = 78.06, *p* < 0.0001), and V_mp_ (F(3, 16) = 72.25, *p* < 0.0001) also showed significant overall effects, but in each case PSE/TO and ZTO/TO were not significantly different from each other (a < b = b < c), while both differed significantly from TO and from PSE/ZTO/TO. This suggests that dye sensitization alone and heterojunction formation alone contribute comparably to the voltage-related descriptors, whereas their effects on current-related descriptors are distinguishable, with the combined configuration (PSE/ZTO/TO) providing a further significant improvement across all parameters (Table 1).

**Figure 8:**
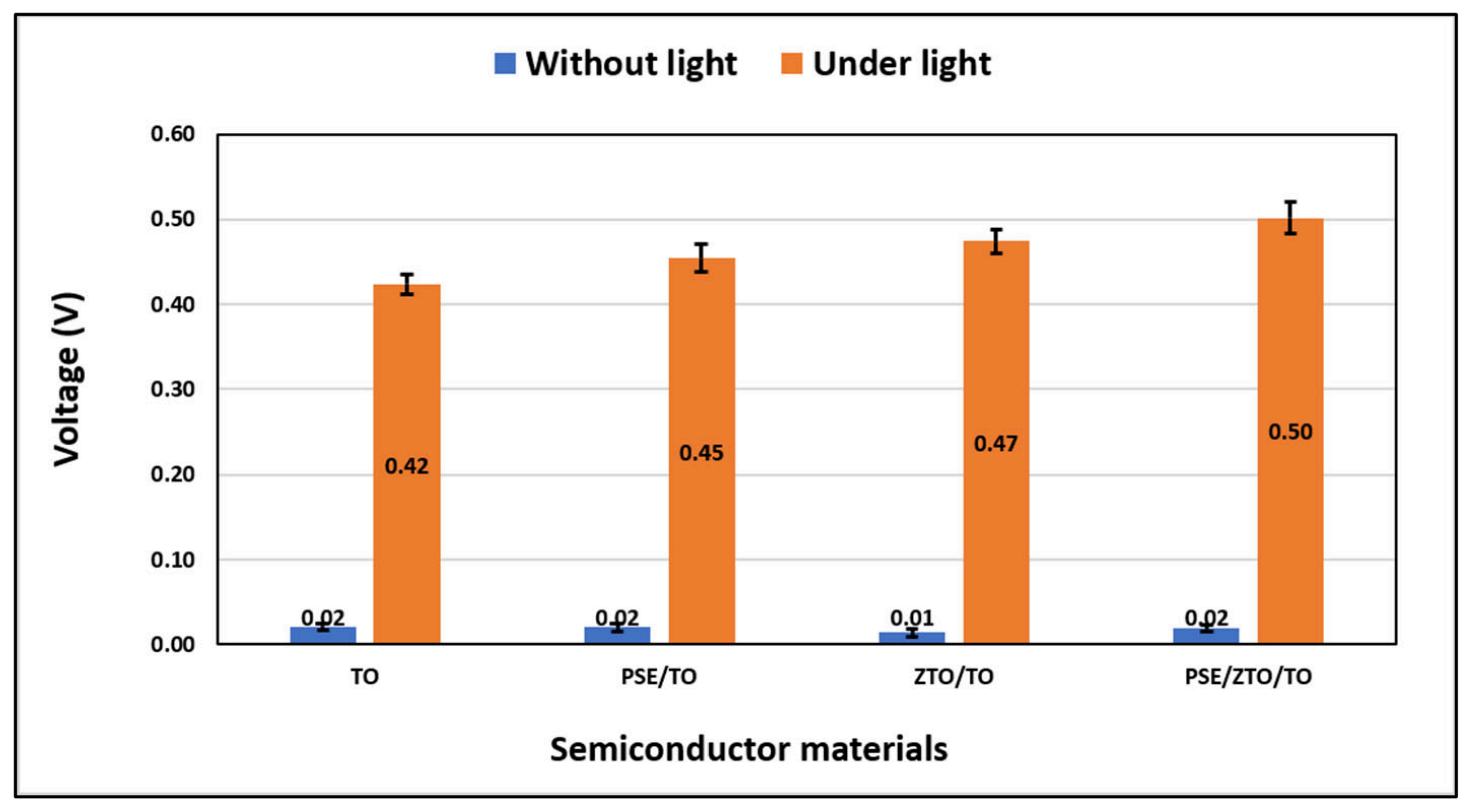
Average open-circuit voltage values in the studied cells.

Moreover, Figure 8 shows the average open-circuit voltage values for each of the evaluated cells. As the figure shows, the ZnTiO_3_/TiO_2_ cell sensitized with dye extracted from *Pycnoporus sanguineus* exhibited the highest energy efficiency. In general, the presence of the dye improves the performance of both the TiO_2_ and the ZnTiO_3_/TiO_2_ heterojunction.

### 2.4. Computational Study

**Figure 9:**
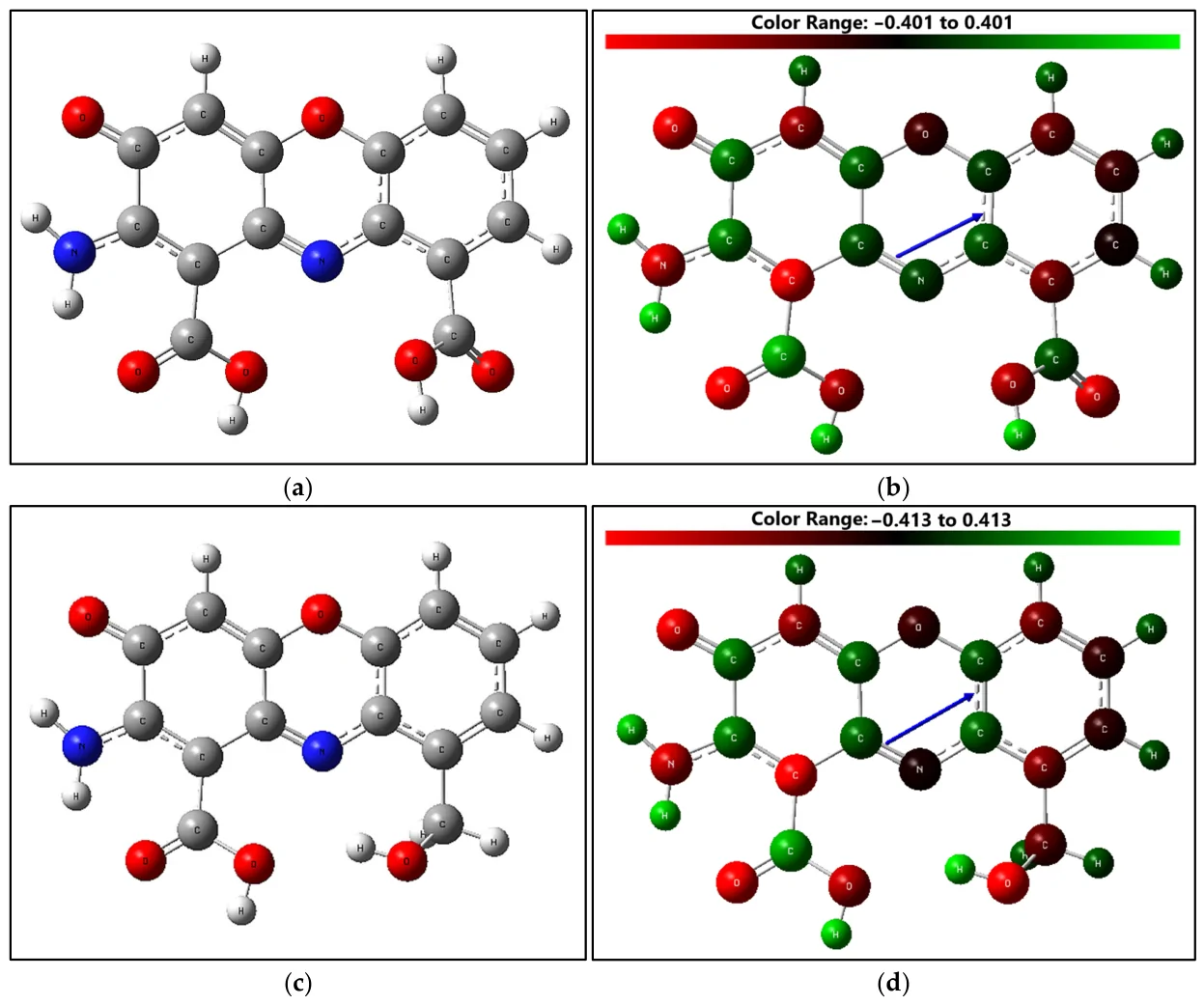
(**a**) Molecular structure and (**b**) dipole moment of cinnabarinic acid, and (**c**) molecular structure and (**d**) dipole moment of cinnabarin. The blue arrow indicates the direction of the molecular dipole moment vector.

The optimization and calculation of the electronic structure of ZTO and TO were reported in a previous study [42]. On the other hand, the cinnabaric acid and cinnabarin structures were optimized using a DFT formalism/UB3LYP method with the Alhrich/TZVP basis sets. Figure 9 shows the optimized structures and their respective dipole moments.

The Gaussian calculation summaries for cinnabarinic acid and cinnabarin structures are compared in Table 2.

The energy positions of the conduction band edge (E_CB_) and valence band edge (E_VB_) for the investigated compounds were theoretically estimated using the empirical absolute electronegativity model proposed by Butler and Ginley, according to the following equations [43]:

(2) $$E_{CB}=\chi -E_{C}-0.5E_{g}$$

(3) $$E_{VB}=E_{CB}+E_{g}$$

where χ is the absolute electronegativity of the compound, and E_g_ represents the corresponding energy band gap. The term E_C_ represents the energy of free electrons on the hydrogen scale, for which the standard constant value of 4.5 eV relative to the vacuum level was adopted. All calculated electronic potentials are reported herein with respect to the absolute vacuum scale, where negative values for E_CB_ denote bound electronic states lying beneath the free electron reference level (E = 0 eV).

**Figure 10:**
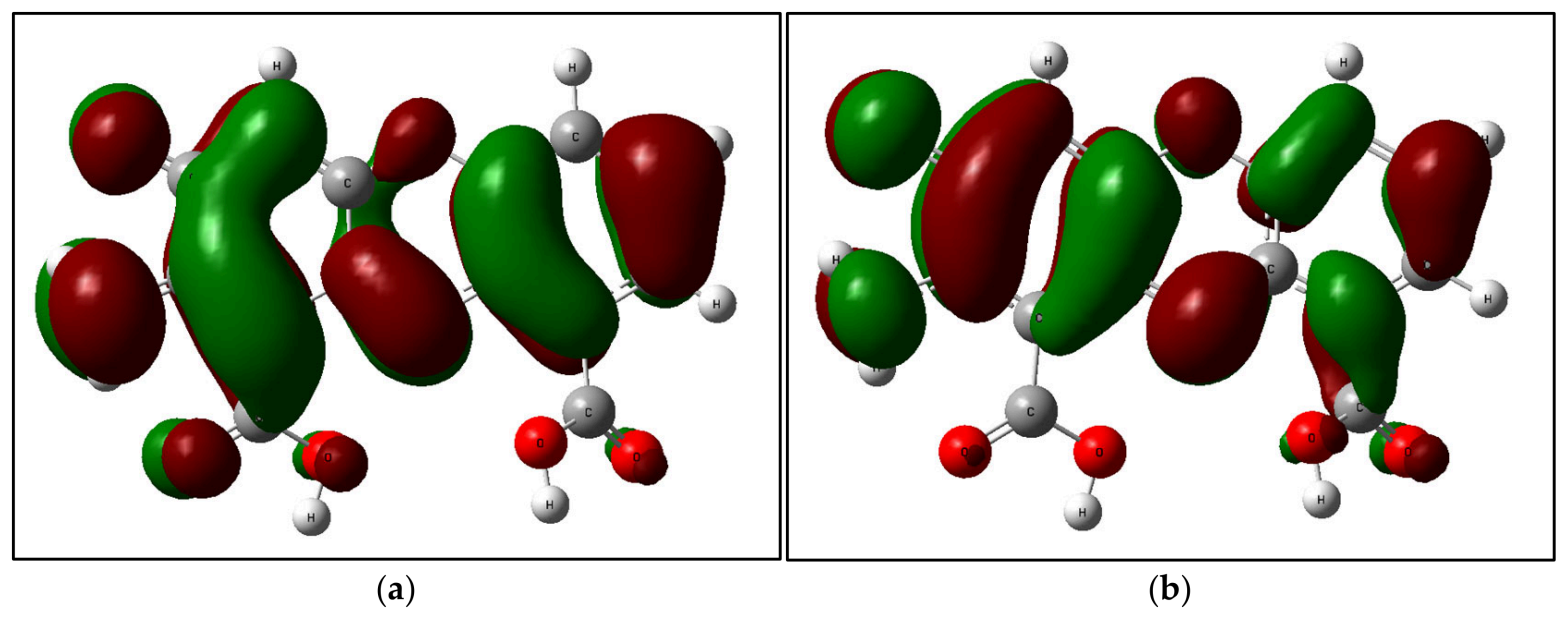

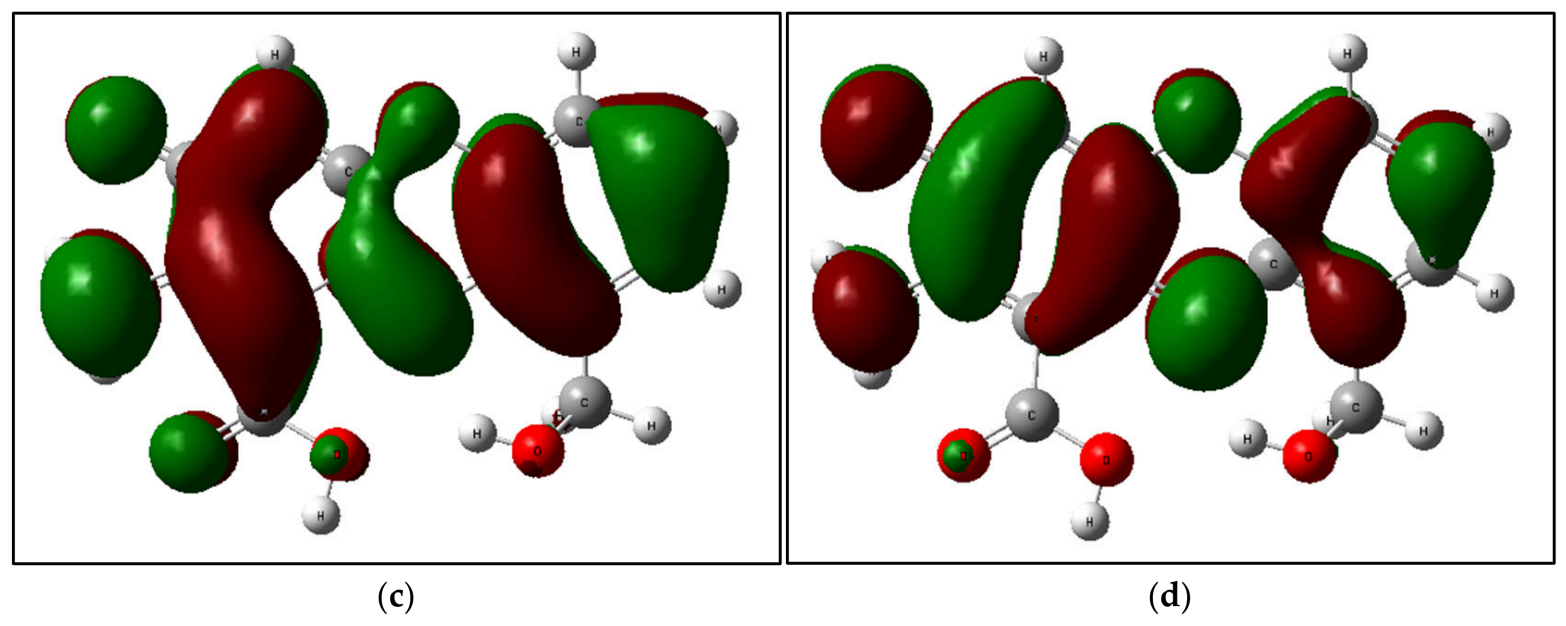
Molecular boundary orbitals: (**a**) HOMO and (**b**) LUMO of cinnabaric acid, and (**c**) HOMO and (**d**) LUMO of cinnabarin.

The distribution and energy levels of the frontier molecular orbitals of cinnabarinic acid and cinnabarin structures are shown in Figure 10.

**Figure 11:**
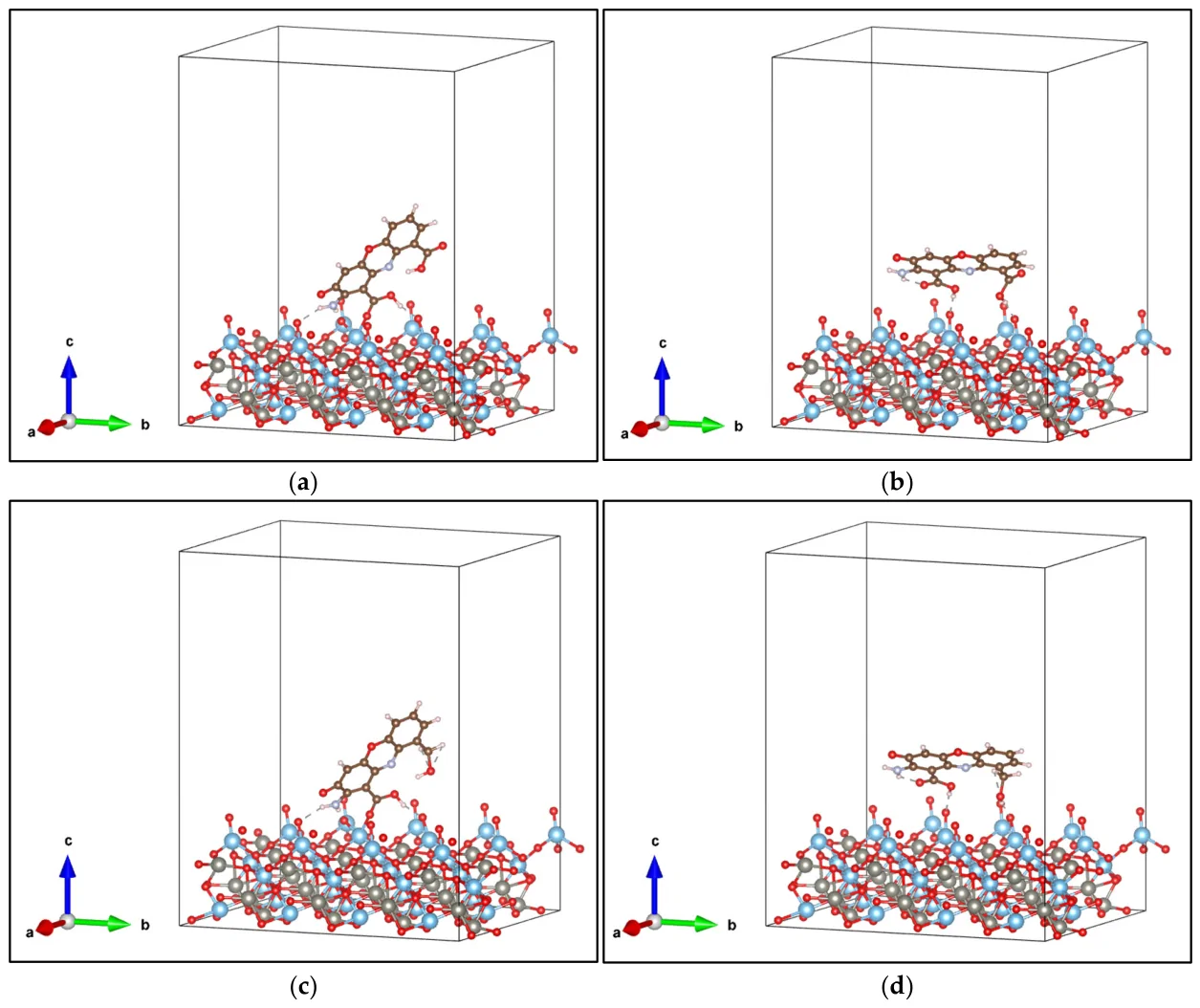
Cinnabaric acid molecule in (**a**) vertical and (**b**) horizontal orientations, and cinnabarin molecule in (**c**) vertical and (**d**) horizontal orientations on the surface (101) of ZnTiO_3_. Color representation: pink spheres represent hydrogen atoms (H), blue spheres represent titanium atoms (Ti), gray spheres represent zinc atoms (Zn), red spheres represent oxygen atoms (O), and white spheres represent nitrogen atoms (N).

**Figure 12:**
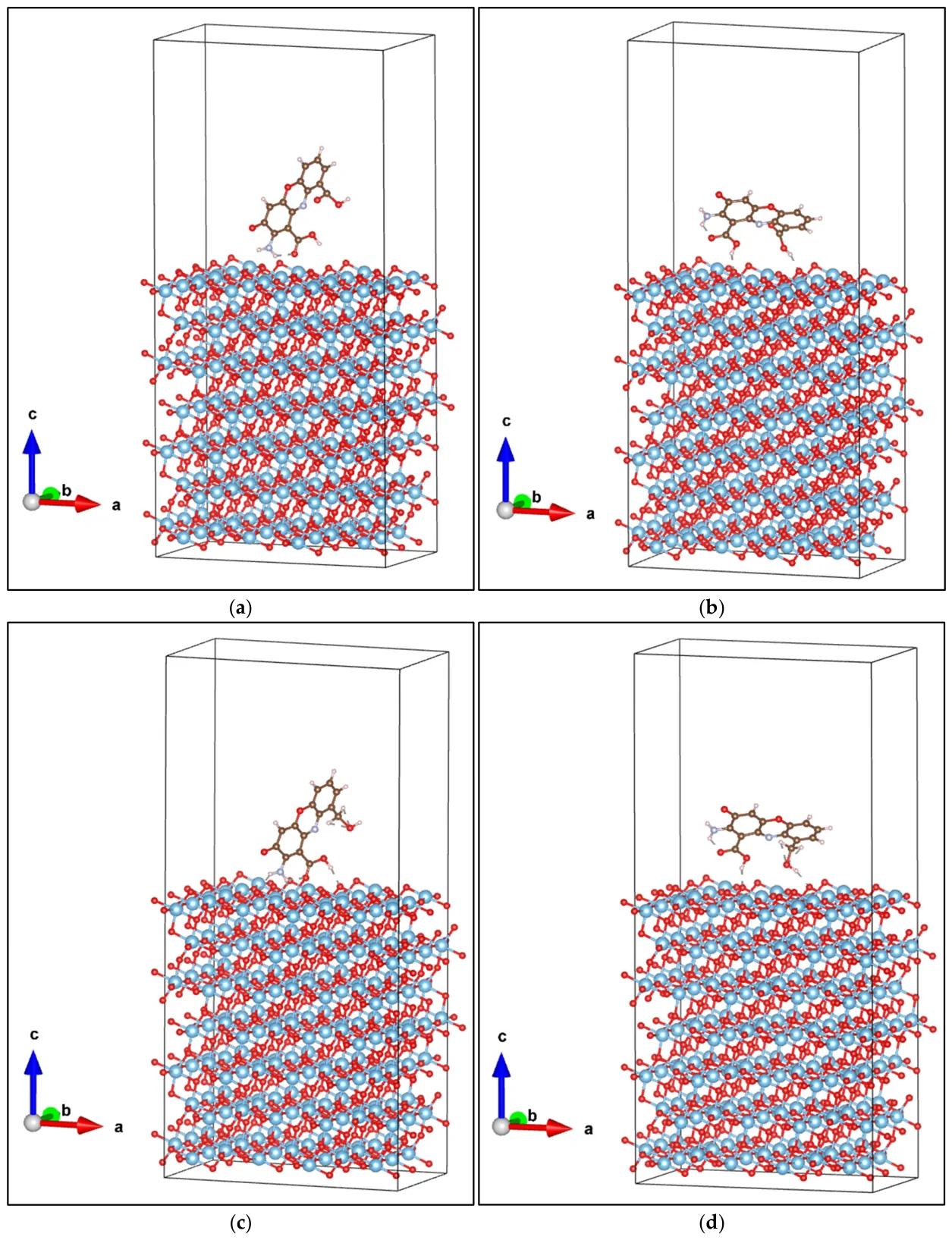
Cinnabaric acid molecule in (**a**) vertical and (**b**) horizontal orientations, and cinnabarin molecule in (**c**) vertical and (**d**) horizontal orientations on the surface (101) of TiO_2_. Color representation: pink spheres represent hydrogen atoms (H), blue spheres represent titanium atoms (Ti), gray spheres represent zinc atoms (Zn), red spheres represent oxygen atoms (O), and white spheres represent nitrogen atoms (N).

Regarding the adsorption of cinnabarinic acid and cinnabarin on the surfaces (101) of both ZnTiO_3_ (ZTO) and TiO_2_ (TO), Figure 11 and Figure 12 show the tested models. Figure 11a,b show the cinnabarinic acid molecule on the surface of ZTO in both vertical (V) and horizontal (H) orientations, respectively, while the cinnabarin molecule on this surface in both vertical (V) and horizontal (H) orientations is shown in Figure 11c and Figure 11d, respectively.

Similarly, Figure 12a,b show the cinnabarinic acid molecule on the surface of TO in both vertical (V) and horizontal (H) orientations, respectively, while the cinnabarin molecule on this surface in both vertical (V) and horizontal (H) orientations is shown in Figure 12c and Figure 12d, respectively.

The adsorption of the cinnabarinic acid molecule was energetically more favorable on the (101) surface of ZTO (E_ads_ = −206.07 kJ/mol), while the adsorption of the cinnabarin molecule was energetically more favorable on the (101) surface of TO (E_ads_ = −251.82 kJ/mol). In both cases, the vertical orientation of the molecule with respect to the semiconductor surface proved to be the most stable. Table 3 shows the adsorption energies of the dye molecules on the semiconductor surfaces in both horizontal and vertical orientations. Table S1 shows the bonds and interaction distances between cinnabaric acid and cinnabarin molecules and the semiconductors ZnTiO_3_ and TiO_2_.

## 3. Discussion

### 3.1. Compositional Basis of the Sensitizer Extract

The HPLC-DAD profile of the *Pycnoporus sanguineus* extract (Figure 1) shows UV maxima at 233 and 430 nm with chromatographic peaks between 9.5 and 10 min. The 430 nm band is indicative of an extended phenoxazine chromophore, whose conjugated tricyclic ring system places the π→π* and n→π* transitions in the visible range, precisely the spectral region that a DSSC sensitizer must cover to complement the intrinsically UV absorption of TiO_2_ and ZnTiO_3_ (forbidden bands of 3.21 and 3.16 eV, respectively [42]). According to the literature, the absorption of visible light through the conjugated core, combined with peripheral carbonyl/hydroxyl anchoring groups, is precisely the structural model required for any DSSC sensitizer, whether natural or synthetic [4,5]. Direct UV-Vis and PL measurements of the extract (Figure 2) confirm this light-harvesting behavior experimentally: the 430 nm absorption band overlaps the emission range of the light source used (Figure S2), while the weak PL signal is consistent with the low fluorescence quantum yield expected for this phenoxazinone-dicarboxylic acid chromophore family [37]. A molecule that only absorbed light without possessing a means to chemically bind to the semiconductor surface would be photoactive but functionally inert as a sensitizer, since the efficiency of electron injection increases with the strength of electronic coupling at the dye-semiconductor interface [44,45].

Additionally, the FTIR spectrum (Figure 3) allowed the identification of functional groups that could participate in the binding of the molecules to the semiconductor surface. Among the main signals identified were the following: the broad hydroxyl/amino stretching band at 3500–3200 cm^−1^, the carbonyl (C=O) stretching band at 1730 cm^−1^ attributable to the carboxyl group of cinnabaric acid, and the C=C/C=N ring vibrations at 1582 and 1538 cm^−1^ characteristic of the conjugated phenoxazine core. Therefore, based on these results, it is possible to suggest that cinnabaric acid and cinnabarin are the main chromophores present in the *Pycnoporus sanguineus* extract, consistent with reports from other authors [13]. Compared to the broader literature on fungal pigments, the spectroscopic profile of *Pycnoporus sanguineus* is structurally distinct from previously reported fungal DSSC sensitizers. The crude *Cortinarius* extracts evaluated by Zalas et al. (2015) [23] and the fungal melanins evaluated by Valdés-Santiago et al. (2023) [24] are both chemically heterogeneous: *Cortinarius* pigments comprise a mixture of anthraquinone-like compounds, while melanins are irregular, high-molecular-weight polymers with broad and poorly defined adsorption. Cinnabarin and cinnabaric acid, on the other hand, are two structurally well-defined, low-molecular-weight phenoxazines with a single dominant chromophore and a specific, identifiable anchoring chemistry. This distinction is important from a mechanistic point of view since a well-defined molecular structure allows electronic structure calculations to be interpreted unambiguously, in a way that would not be possible for a heterogeneous crude extract or a polymeric pigment [21,22] and represents a methodological advantage of this fungal system over previous fungal DSSC studies.

### 3.2. Structural Features of the ZnTiO_3_/TiO_2_ Heterostructure Relative to TiO_2_

X-ray diffraction (XRD) confirms the coexistence of hexagonal ZnTiO_3_ (space group R-3(148), ICDD 00-015-0591) with anatase-phase TiO_2_ (and small amounts of rutile) in the ZTO/TO sample, compared to a single anatase phase in the TO sample (Figure 4). The predominance of the anatase phase over the rutile phase is functionally significant because the anatase phase has a more negative conduction band edge and greater electron mobility than rutile, characteristics that favor electron injection and transport in the photoanodes of dye-sensitized solar cells (DSSCs). For this reason, it is consistently identified as the preferred TiO_2_ polymorph in the DSSC literature [5,28].

The Scherrer crystallite size is 27.51 ± 1.27 nm for the ZnTiO_3_ phase and 43.27 ± 1.52 nm for the anatase TiO_2_ phase. The relatively small crystallite size obtained for the ZnTiO_3_ phase is consistent with the sol–gel synthesis route used here, which has been reported to yield smaller zinc titanate crystallites than thermal- or plasma-assisted synthesis routes [46]. SEM shows larger particles for TO (~50 nm) than for ZTO/TO (~38 nm) (Figure 5), consistent with the BET specific surface area, which is higher for ZTO/TO (87.6 m^2^/g) than for TO (81.4 m^2^/g). This higher surface area of ZTO/TO relative to TO is consistent with the improved photovoltaic performance observed for the heterojunction devices, together with the electronic advantages of the ZnTiO_3_/TiO_2_ junction described below.

### 3.3. Photovoltaic Performance and Statistical Validation

The J-V and P-V curves (Figure 6 and Figure 7) and the summary in Table 1 show a consistent improvement in the six photovoltaic descriptors defined by the standard DSSC parameter framework [4] as each design feature is added: V_oc_ increases from 0.42 ± 0.01 V (TO) to 0.45 ± 0.02 V (PSE/TO) to 0.47 ± 0.01 V (ZTO/TO) to 0.50 ± 0.02 V (PSE/ZTO/TO); J_sc_ increases from 0.28 to 1.14 mA/cm^2^; FF increases from 0.48 to 0.67; and η increases from 0.06% to 0.38% in the same series. The fact that each descriptor moves in the same direction and order (TO < PSE/TO < ZTO/TO < PSE/ZTO/TO) suggests that sensitization and heterostructure engineering could solve the problem of energy loss in the process, although not through the same mechanism.

Unlike V_oc_ and J_sc_, the fill factor (FF) is governed primarily by series and parallel resistances, and by open-circuit and on-load recombination kinetics, rather than directly by light absorption or band-edge position [4]. The increase in fill factor (FF) from 0.48 (TO) to 0.67 (PSE/ZTO/TO) suggests improved charge transfer and reduced resistance to interfacial recombination in the complete device, a pattern consistent with the lower charge transfer resistance and higher fill factors observed when introducing analogous heterojunction/doping strategies in TiO_2_ photoanodes in other studies, for example, the increased recombination resistance measured by impedance spectroscopy after Ni doping of TiO_2_ [29], and the lower electron transport resistance observed in TiO_2_/g-C_3_N_4_ heterostructure photoanodes compared to pure TiO_2_ [30].

Finally, one-way ANOVA (*n* = 5 per configuration) confirmed a statistically significant treatment effect on all six photovoltaic descriptors (Table 1). Post hoc Tukey/Duncan tests separated J_sc_, J_mp_, and η into four fully distinct groups (a < b < c < d), whereas V_oc_, FF, and V_mp_ placed PSE/TO and ZTO/TO in the same statistical group (a < b = b < c). The absolute PCE achieved by the best-performing configuration (0.38%, PSE/ZTO/TO) remains modest relative to synthetic and plant-derived sensitizers (Table 4), and this gap is even more pronounced against crystalline silicon photovoltaics (>20%) and high-efficiency ruthenium-based DSSCs (up to ~12% under optimized conditions). Nonetheless, the statistically validated, consistent improvement across all six photovoltaic descriptors demonstrates that both dye sensitization and heterojunction engineering contribute robustly and reproducibly to device performance, underscoring that the present contribution lies in establishing a novel, mechanistically characterized fungal sensitizer class rather than in achieving competitive absolute efficiency at this stage.

### 3.4. Predicted Band-Edge Alignment Rationalizes the Electron-Transfer Pathway

**Figure 13:**
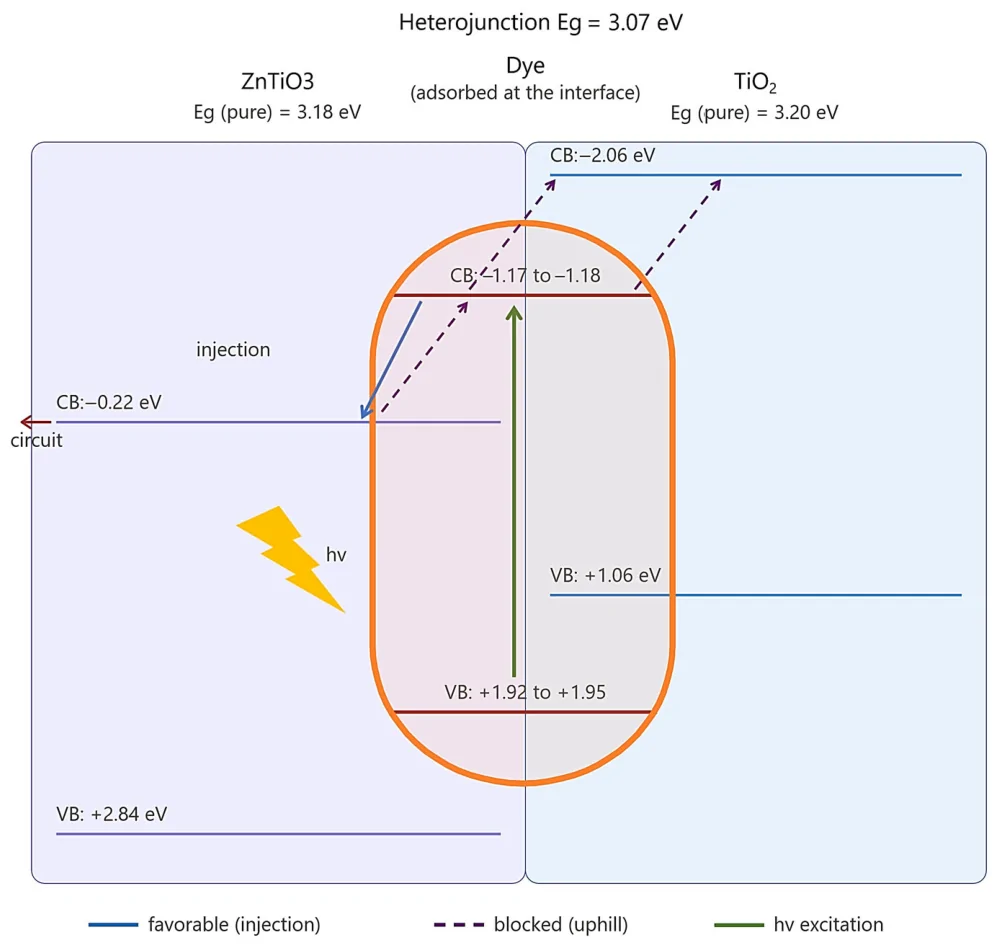
Electron injection pathway from the dye fraction adsorbed on the TiO_2_ surface within the ZnTiO_3_/TiO_2_ heterojunction.

Table 2 shows the E_CB_ and E_VB_ values for cinnabaric acid (E_CB_ = −1.18 eV, E_VB_ = +1.92 eV) and cinnabarin (E_CB_ = −1.17 eV, E_VB_ = +1.94 eV), calculated using the Butler–Ginley model (Equations (2) and (3)). These positions can be directly compared with the E_CB_ and E_VB_ values reported in a previous study for the two semiconductor phases of the photoanode: ZnTiO_3_ (E_CB_ = −0.22, E_VB_ = +2.84 eV) and TiO_2_ (E_CB_ = −2.06, E_VB_ = +1.06 eV). Unlike the qualitative picture usually obtained from HOMO/LUMO or electronegativity trends, this direct comparison places the dye levels on the same absolute vacuum scale as the oxides, allowing the driving force of electron injection to be quantified. On this scale, ZnTiO_3_ (−0.22 eV) is the least reducing conduction band and TiO_2_ (−2.06 eV) is the most reducing, with the E_CB_ values for both dyes located between them, near the midpoint but shifted towards the TiO_2_ end. This places the injection of dye electrons into the ZnTiO_3_ conduction band in a thermodynamically favorable, downward state—approximately 0.955 eV for cinnabaric acid and 0.945 eV for cinnabarin—whereas direct injection into the TiO_2_ conduction band would require an upward energy gain of approximately 0.885–0.895 eV for both dyes, and is therefore thermodynamically unfavorable (Figure 13). This agrees with DFT-level injection rate calculations reported in the literature for dyes adsorbed on ZnTiO_3_, where the injection efficiency reflects the energy proximity between the excited state of the dye and the ZnTiO_3_ conduction band [35].

Cinnabarin shows a strong preference for selective chemisorption onto TiO_2_ (−251.82 kJ/mol, vertical) over ZnTiO_3_, whereas cinnabaric acid adsorbs comparably onto both oxides, with only a slight preference for ZnTiO_3_ (−206.07 kJ/mol vs. −201.40 kJ/mol for TiO_2_, a difference of approximately 2%). Since TiO_2_ is the thermodynamically unfavorable injection target, cinnabaric acid—which chemisorbs onto ZnTiO_3_ almost as readily as onto TiO_2_—is well-positioned to directly exploit the energetically preferred ZnTiO_3_ channel. Cinnabarin, in contrast, adsorbs predominantly to sites on the TiO_2_ surface where direct injection into the TiO_2_ conduction band is unfavorable by ~0.9 eV. Therefore, its contribution to photocurrent generation likely occurs through interfacial contact with adjacent ZnTiO_3_ domains within the heterojunction or through the ultrafast (femtosecond-scale) injection kinetics characteristic of dye-sensitized systems, which can partially overcome modest thermodynamic penalties of this magnitude. Taken together, these results define a ZnTiO_3_-centered injection pathway, fueled by both dyes, but more directly by cinnabaric acid.

Once electrons occupy the conduction band of ZnTiO_3_, they cannot advance to the higher-level conduction band of TiO_2_, as this transition would require an energy gain of ~1.84 eV (the E_CB_ shift between the two phases: −2.06 − (−0.22) eV). Therefore, TiO_2_ acts as an energy barrier that confines the injected carriers within the ZnTiO_3_ lattice, functioning as a one-way gate against backtransfer and associated interfacial recombination pathways, rather than as a two-way conduit. This stepped (type II) band-shifting mechanism parallels several reported photoanode strategies that increase V_oc_ via the same route: Ni-doped TiO_2_/TiO_2_ homojunctions, where Mott–Schottky analysis showed a more negative flat band potential and longer electron lifetime after doping [29]; TiO_2_/g-C_3_N_4_ heterostructures, where the g-C_3_N_4_ layer was explicitly identified as a recombination-blocking layer [30]; and photoanode designs with cascaded energy levels, where an upward shift in the TiO_2_ Fermi level, engineered using perovskite, suppressed the potential barrier responsible for voltage loss at the transparent conductor/TiO_2_ interface [47]. The classic basis for this type of argument—that the V_oc_ in nanocrystalline TiO_2_-based DSSCs is governed by the position of the conduction band edge relative to the recombination rate at that edge—was established by intensity-modulated photovoltage and photocurrent spectroscopy [26,27].

Figure 13 is directly supported by the experimental data in Table 1: the V_oc_ gain of +0.05 V measured for ZTO/TO over pure TO occurs even in the total absence of dye, ruling out any explanation based on improved light absorption or electron injection and leaving recombination suppression as essentially the only mechanism consistent with both the band-alignment calculation and the dye-free device data. There is also a direct precedent for this specific chemistry: a reduced graphene-oxide ZnTiO_3_ photoanode outperformed unmodified ZnTiO_3_ due to a lower density of surface trap states and better electron transport [33], supporting the premise that ZnTiO_3_-based heterojunctions act primarily through trap/interfacial state effects rather than through increased light absorption. This is supported by considering the band gaps of the conjugated versus the independent phases. The literature shows that the experimentally measured band gap for the ZnTiO_3_/TiO_2_ heterojunction is 3.07 eV, while the band gaps of the pure phases are somewhat larger: 3.18 eV for ZnTiO_3_ and 3.20 eV for TiO_2_ [42,48]. This represents a modest and expected reduction between the heterojunction and pure phase values, consistent with interfacial coupling effects at the ZnTiO_3_/TiO_2_ boundary. In all cases, the oxides remain wide-bandgap semiconductors that are active only in the UV region (E_g_ ≈ 3.07–3.21 eV), reinforcing the idea that the contribution of the ZnTiO_3_ layer to the device performance is electronic (band-edge engineering/trap states) rather than related to any extension of visible light absorption.

### 3.5. Adsorption Configuration

The adsorption energies calculated using DFT (Table 3) show that, for both dyes and on both surfaces, the vertical orientation is significantly more stable than the horizontal, with a difference of approximately 2.5 to 5.6 times. Table S1 provides the structural explanation: in the horizontal configuration, both dyes bind to each surface exclusively via hydrogen bonds (2 H bonds per system, 1.63–1.94 Å). In the vertical configuration, this network of hydrogen bonds is maintained, but it is complemented by an additional, shorter O–Ti bond (2.03–2.13 Å, for example, O_113_–Ti_17_ on ZTO and O_341_/O_113_–Ti_57_ on TO), which falls within the typical range of a dative covalent metal-oxygen coordination bond, rather than a hydrogen bond. From a mechanistic perspective, the rotation of the anchoring group from a parallel (horizontal) to a perpendicular (vertical) orientation brings a deprotonated carbonyl oxygen or hydroxyl group close enough to a Ti^4+^ center on the surface to form a genuine dative bond, transforming what was a purely physisorption hydrogen-bonding contact into a mixed chemisorption and hydrogen-bonding interface. Since covalent and dative-covalent bonds are intrinsically stronger than hydrogen bonds, this single additional bond is likely the reason for the vertical/horizontal stability difference. This chemisorption pathway reflects the mechanism described in the literature for other natural dyes anchored to TiO_2_. For example, molecular dynamics simulations have shown that cyanidin-3-glucoside and bixin chemisorb onto TiO_2_ via sequential deprotonation and the formation of Ti-O bonds through monodentate hydroxyl and carboxyl anchoring groups, respectively [45]. Similarly, the general principle that carboxylic/benzoic acid derivatives bind directly and reproducibly to TiO_2_ surfaces was established in early work on hybrid DSSC devices [44]. This also agrees with the DFT modeling of other chromophores adsorbed on TiO_2_, where it was shown that the distinction between monodentate and bidentate anchoring modes substantially modifies the adsorption stability and, by extension, the electron injection efficiency [34].

In addition to the shared vertical orientation mechanism, both pigments exhibit distinct surface preferences. Cinnabaric acid preferentially adsorbs to ZnTiO_3_ (−206.07 kJ/mol, vertical), while cinnabarin preferentially adsorbs to TiO_2_ (−251.82 kJ/mol, vertical). This comparison was deliberately limited to the amino and carboxyl groups located at the common end of both molecules, rather than to the substituent at the opposite end of the phenoxazine core: the additional carboxyl group unique to cinnabaric acid or the hydroxyl group unique to cinnabarin. Evaluating adsorption at that non-shared distal end would have yielded a predictable result: carboxylic acid groups are well established as strong, directly chemisorbed anchors on TiO_2_-type surfaces [44], while hydroxyl groups are typically weaker anchors. Therefore, any preference measured there would simply reflect the known hierarchy of anchoring group chemistry rather than some intrinsic property of the two dyes being compared. By keeping the anchoring chemistry constant—analyzing the amino/carboxyl end common to both molecules—the comparison isolates a truly informative variable: whether the distal substituent, despite not being in contact with the surface, modulates the binding behavior of the shared anchoring group. Structurally, both dyes anchor to either surface through this common end. The additional carboxyl group of cinnabaric acid and the methyl group of cinnabarin, both located at opposite ends of the molecule, do not directly participate in surface interaction in either configuration. Therefore, the differentiated surface preference must originate from an electronic effect rather than a structural/anchoring point effect: although the distal substituent does not directly bind to the surface, it is conjugated through the phenoxazine ring system and can inductively and resonantly modulate the electron density at the shared amino/carboxyl anchoring end. The electron-withdrawing carboxyl group of cinnabaric acid would be expected to reduce the electron density at this end relative to the electron-donating methyl group of cinnabarin, a difference consistent with the higher dipole moment calculated for cinnabaric acid (2.50 vs. 1.87 Debye, Table 2). This could favor stronger electrostatic complementarity with the more heterogeneous Zn^2+^/Ti^4+^ surface of ZnTiO_3_, while the electron-rich, less polarized anchoring end of cinnabarin might favor the more uniform Ti^4+^ network of TiO_2_. This interpretation is consistent with the general finding that substituents beyond the anchoring group itself can modulate the binding affinity in dye series bonded to TiO_2_ [34].

### 3.6. Comparison with Grätzel Cells with Natural Dyes Reported in the Literature

In this study, four configurations were comparatively evaluated: TO, PSE/TO, ZTO/TO, and PSE/ZTO/TO. An improvement was demonstrated in the fully sensitized PSE/ZTO/TO heterostructure compared to pure TiO_2_. This improvement could be due to a combination of effects, such as dye sensitization (PSE/TO vs. TO) and heterostructure generation (ZTO/TO vs. TO). In fact, the V_oc_, J_sc_, FF, and η values shown in Table 1 exhibit a consistent and increasing pattern of positive interaction. Furthermore, a comparison was made between the results of this study and those reported by other authors. Table 4 compiles the open-circuit voltage (V_oc_), short-circuit current density (J_sc_), fill factor (FF), and power conversion efficiency (η) of 19 solar cells sensitized with natural plant-derived dyes and 6 solar cells sensitized with fungal dyes, using pigments from *Cortinarius* [23] along with the PSE/ZTO/TO system from this work. Compared to the fungal subset, the PSE/ZTO/TO system developed here suggests that phenoxazine pigments extracted from *Pycnoporus sanguineus* represent a competitive contribution to the literature on dye-sensitized solar cells (DSSCs) of fungal origin.

**Table 4:** Photoelectrochemical performance parameters (V_oc_, J_sc_, FF, η) of natural-dye-sensitized solar cells reported in the literature, compared with the present work.

Source	Type	V_oc_ (V)	J_sc_ (mA/cm^2^)	FF	η (%)	Reference
N-719 (Ru reference dye)	Synthetic (reference)	0.77	13.74	0.58	6.11	[49]
Mangosteen pericarp	Plant	0.69	2.69	0.63	1.17	[49]
Spinach-derived modified chlorophyll/β-carotene	Plant	0.53	13.70	0.58	4.20	[50]
*Opuntia ficus-indica* (Sicilian prickly pear)	Plant	0.39	8.80	0.60	2.06	[51]
*Beta vulgaris rubra* (Red turnip)	Plant	0.43	9.50	0.37	1.70	[52]
*Canarium odontophyllum + Ixora* sp. (mixed ethanolic extract)	Plant	0.38	6.26	0.47	1.13	[53]
*Ixora* sp.	Plant	0.35	6.26	0.44	0.96	[53]
*Canarium odontophyllum*	Plant	0.39	2.45	0.62	0.59	[53]
*Canarium odontophyllum + Ixora* sp. (consecutive layers)	Plant	0.34	9.80	0.46	1.55	[53]
*Rhoeo spathacea (Sw.) Stearn* (water-based DSSC)	Plant	0.50	10.90	0.27	1.49	[54]
Bixin	Plant	0.57	1.10	0.59	0.37	[55]
Annatto	Plant	0.56	0.53	0.66	0.19	[55]
Norbixin	Plant	0.53	0.38	0.64	0.13	[55]
*Cortinarius sanguineus*	Fungal	0.54	1.79	0.66	0.64	[23]
*Cortinarius semisanguineus* (Css1)	Fungal	0.54	1.56	0.63	0.54	[23]
*Cortinarius semisanguineus* (Css2)	Fungal	0.54	1.52	0.55	0.45	[23]
*Cortinarius* sp. *(sect. Telamonia)*	Fungal	0.53	1.10	0.64	0.37	[23]
*Cortinarius croceus*	Fungal	0.48	0.98	0.64	0.30	[23]
*Cortinarius* sp. *(sect. Dermocybe)*	Fungal	0.48	0.88	0.62	0.26	[23]
*Pycnoporus sanguineus* (PSE/ZTO/TO)	Fungal	0.50	1.14	0.67	0.38	This study
*Pycnoporus sanguineus* (PSE/TO)	Fungal	0.47	0.74	0.55	0.19	This study

## 4. Materials and Methods

### 4.1. Materials

All reagents were purchased from commercial sources and used without further purification: Isopropyl alcohol [C_3_H_8_O] (Sigma Aldrich, St. Louis, MO, USA, ≥99.5%), Titanium(IV) isopropoxide [Ti(OC_3_H_7_)_4_] (Sigma Aldrich, St. Louis, MO, USA, 98%), Zinc acetate dihydrate [Zn(CH_3_COO)_2_·2H_2_O] (ACS, St. Louis, MO, USA, ≥98%), Potato Dextrose Agar (Merck KGaA, Darmstadt, Germany), Potassium iodide [KI] (Merck KGaA, Darmstadt, Germany, ACS reagent, ≥99.0%), Iodine [I_2_] (Merck KGaA, Darmstadt, Germany, ACS reagent, ≥99.8%), and Ethylene glycol [C_2_H_6_O_2_] (Merck KGaA, Darmstadt, Germany, ACS reagent, ≥99.5%). ITO-coated conductive glasses (resistivity: <10 ohm/sq and light transmittance: >84%) were purchased from Dongguan Qunguan Electronic Technology Co. Ltd., Dongguan, China. The UV-Vis absorbance spectrum of the *Pycnoporus sanguineus* extract was recorded using a PerkinElmer LAMBDA 365+/1050+ spectrophotometer (PerkinElmer, Shelton, CT, USA) in the 200–900 nm range. The photoluminescence (PL) emission spectrum was recorded using an Edinburgh Instruments FLS1000 spectrofluorometer (Edinburgh Instruments, Livingston, UK) at an excitation of 430 nm. For both measurements, the extract was diluted in methanol at a 1:10 (*v*/*v*) ratio using a quartz cuvette, with pure methanol as a blank/reference. The evaluation of solar cells was performed under standard conditions of 100 mW cm^−2^ using a General Lighting Led lamp (General Lighting Electronic Co., Ltd., Zhongshan, China), whose emission spectrum is shown in Figure S2. Cell performance was preliminarily screened using a Truper Mut-39 digital multimeter (Grupo Truper S.A. de C.V., Jilotepec, Mexico); the J–V curves reported in this study were acquired using an Agilent B2900 Series Precision Source/Measure Unit (Agilent Technologies, Santa Clara, CA, USA), applying a linear voltage sweep from 0 V to the open-circuit voltage of each device, divided into 178–201 equally spaced points (step size of approximately 2–3 mV depending on the cell configuration). Computational calculations were performed using the Gaussian version 16 software package (Gaussian, Inc., Wallingford, CT, USA), and the Vienna Ab initio Simulation Package (VASP) version 6.0 (VASP Software GmbH, Vienna, Austria).

### 4.2. Reactivation of the Strain and Production of Solid-State Biomass

**Figure 14:**
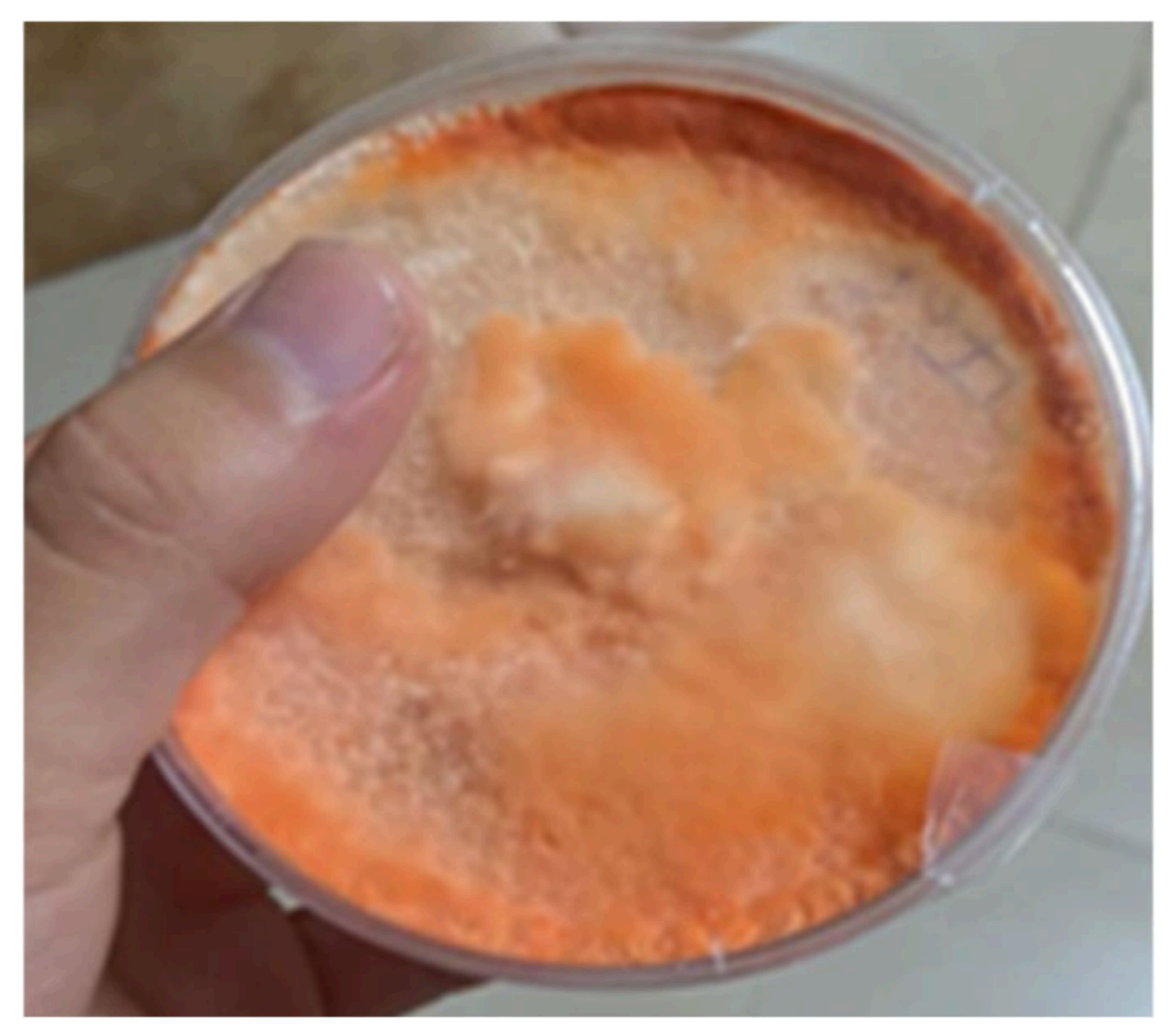
*Pycnoporus sanguineus* strain (EM1990) reactivated in PDA medium, generating biomass.

The *Pycnoporus sanguineus* strain (code EM1990) was provided by the Mycoteca/Fugarium of the Universidad Técnica Particular de Loja [HUTPL(f)]. Reactivation and initial multiplication of the fungus were performed by inoculation onto Potato Dextrose Agar (PDA, Difco Laboratories, Becton Dickinson and Co., Sparks, MD, USA) solid medium, prepared according to the manufacturer’s specifications. The medium was autoclaved at 121 °C and 15 psi for 15 min and then dispensed under aseptic conditions into Petri dishes within a laminar flow hood. The plates were incubated at 27 °C until active mycelial growth and complete colonization of the agar surface were achieved. From these pre-existing cultures, mycelium disks (5 mm in diameter) were taken to inoculate 90 mm diameter Petri dishes containing PDA medium. This solid-state culture system was used to maximize biomass production (Figure 14) for subsequent pigment extraction.

### 4.3. Ultrasound-Assisted Dye Extraction

**Figure 15:**
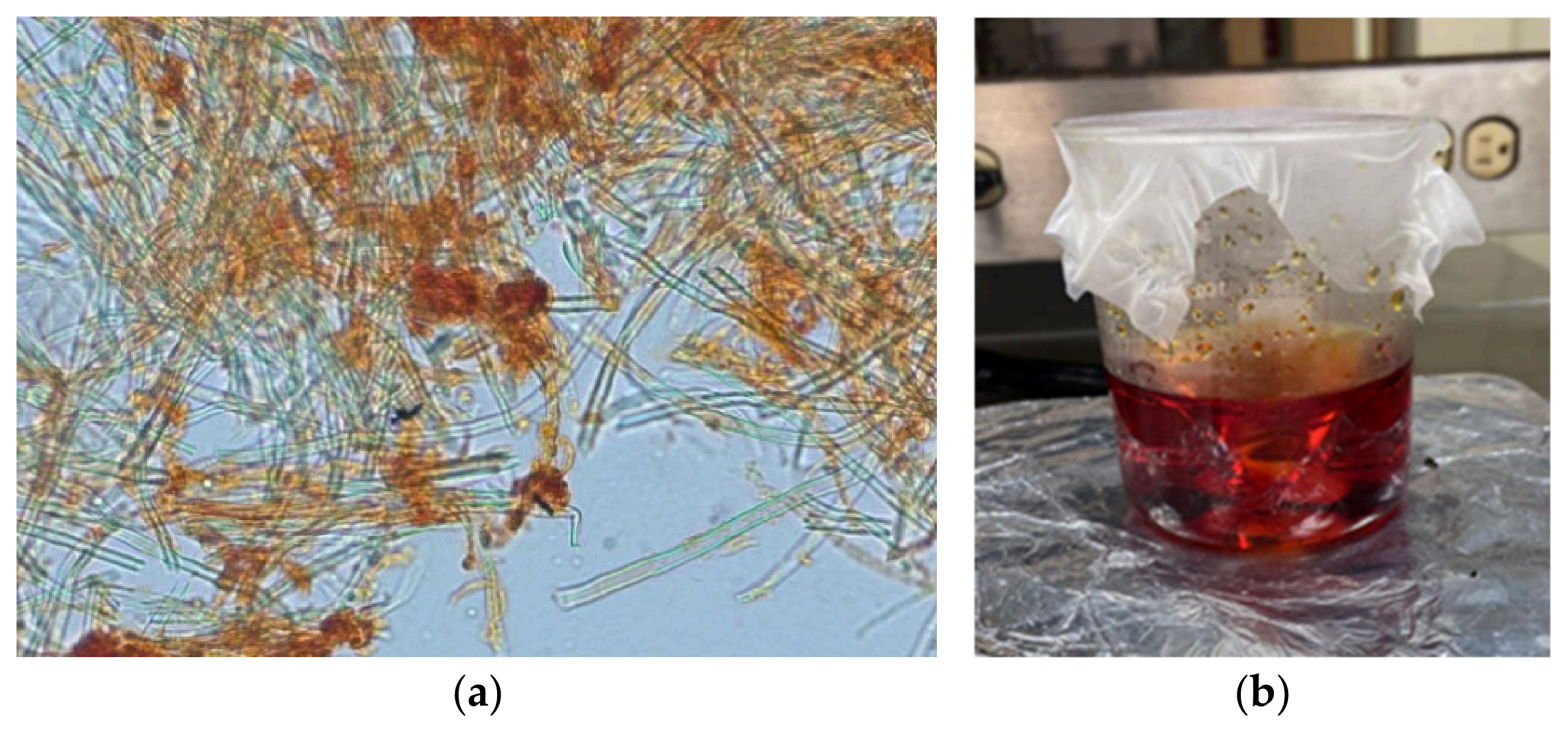
(**a**) Hyphal matrix of the fungus *Pycnoporus sanguineus* obtained after solid-state fermentation; (**b**) hydroalcoholic mixture.

The mycelium was harvested once it covered the entire surface of the medium. Prior to harvesting, light microscopy was used to assess the integrity of the mycelial structure and visually confirm the accumulation and secretion of the characteristic pigment in the culture (Figure 15a). Subsequently, using a sterile scalpel, the surface layer of the mycelial biomass was carefully scraped, minimizing the removal and entrainment of the underlying agar medium. The harvested material was transferred to a drying chamber for three days to ensure complete moisture removal. The dehydrated samples were then pulverized and sieved through a No. 20 mesh to homogenize the particle size. For pigment extraction, the biomass was suspended in a hydroalcoholic mixture (1:1 *v*/*v* ratio) consisting of 110 mL of distilled water and 110 mL of absolute ethanol in a 500 mL flask (Figure 15b). The process was performed by ultrasound-assisted extraction for 30 min at a controlled temperature of 60 °C. Finally, the crude extract was vacuum-filtered to remove insoluble residues, and the resulting filtrate was rotary-evaporated at 35 °C and 240 rpm until complete evaporation of the organic solvent.

### 4.4. Synthesis of ZnTiO3/TiO2 (ZTO/TO) and TiO2 (TO) Semiconductors

The hybrid semiconductor ZnTiO_3_/TiO_2_ (ZTO/TO) was synthesized using a modified sol–gel method as described in previous studies [55]. A 70% *v*/*v* solution of Ti(OC_3_H_7_)_4_ in C_3_H_8_O (Solution A) was prepared. A separate solution B was prepared from Zn(CH_3_COO)_2_·2H_2_O and H_2_O/C_3_H_8_O, using the stoichiometric amount of water required for the hydrolysis of Ti(OC_3_H_7_)_4_ and with a C_3_H_8_O/H_2_O ratio of 50% *v*/*v*. Solution B was then added dropwise to Solution A to obtain a mixture with a ZnO/TiO_2_ molar ratio of 1:3. This mixture was stirred until a white precipitate formed. The precipitate was dried at 60 °C for 24 h and calcined at 500 °C for 4 h. Finally, the products were cooled to room temperature. Similarly, the semiconductor TiO_2_ (TO), in its anatase phase, was synthesized using a modified sol–gel method. First, a 70% *v*/*v* solution of Ti(OC_3_H_7_)_4_ in C_3_H_8_O was prepared. Then, a 50% *v*/*v* aqueous solution of C_3_H_8_O was slowly added. This system was stirred until a white precipitate formed. The precipitate was dried at 60 °C for 24 h and calcined at 500 °C for 4 h. Finally, the products of this synthesis were also cooled to room temperature.

### 4.5. Assembly and Photovoltaic Activity of DSSCs

After synthesizing the ZnTiO_3_/TiO_2_ and TiO_2_ (anatase phase) semiconductors, they were impregnated with isopropyl alcohol to obtain a homogeneous and workable paste. These pastes were deposited onto conductive glass plates coated with ITO to a thickness of approximately 50 microns and allowed to dry slightly to promote adhesion to the glass plate. These plates were then immersed in a dye solution extracted from *Pycnoporus sanguineus* for 24 h. Then, the glass plates with the dye-impregnated semiconductors were rinsed with water to remove excess dye. No loss of the semiconductor layer was observed. Once the glass plates were completely dry, two drops of an electrolytic solution containing an iodide/triiodide (I^−^/I_3_^−^) redox couple were applied. Finally, a counter electrode, consisting of a conductive glass plate coated with graphite, was placed on the glass plate with the dye-impregnated semiconductors. The two glass plates were held one on top of the other using clamps, ensuring a 5 mm offset between them. The dye-sensitized solar cell (DSSC) thus fabricated was sealed on all sides to prevent electrolyte loss. The active (irradiated) area of the assembled cells was 2.25 cm^2^. The performance of solar cells sensitized with the dye extracted from *Pycnoporus sanguineus* was evaluated using characteristic photovoltaic parameters such as: open-circuit voltage (V_oc_, V), short-circuit current density (J_sc_, mA/cm^2^), maximum power (P_max_), current density at maximum power point (J_mp_, mA/cm^2^), filling factor (FF) and efficiency (η, %).

The fill factor (FF) was determined as the ratio between the actual maximum power (P_max_) and the theoretical maximum power (V_oc_ × J_sc_) according to the following equation [56].

(4) $$FF=\frac{P_{max}}{V_{oc}\times J_{sc}}$$

Efficiency, considered as the percentage of solar energy converted into electricity, was determined using the following equation [56].

(5) $$\eta =\frac{P_{max}}{P_{in}}\times 100$$

where P_in_ represents the power density of the incident light (100 mW/cm^2^).

### 4.6. Characterization of Materials

The synthesized semiconductors were characterized by X-ray diffraction (XRD) and scanning electron microscopy (SEM). XRD data were obtained using a Bruker-AXS D8-Discover diffractometer (Bruker AXS, Karlsruhe, Germany) equipped with Cu Kα (1.5406 Å) radiation. The patterns obtained in the 2θ range of 5 to 70° were compared with the ICDD (International Diffraction Data Center, 2018) and COD (Open Crystallography Database, 2018) databases to identify the crystallographic phases. Field-effect scanning electron microscopy (SEM) images were obtained with a Zeiss Gemini ULTRA plus electron microscope (Oberkochen, Germany) operating at 3.0 kV. Samples for SEM measurements were deposited and dried on a silicon wafer. For the characterization of the dye extracted from *Pycnoporus sanguineus* (PS), a Thermo Scientific Dionex Ultimate 3000 high-performance liquid chromatograph (Waltham, MA, USA) with a digital array detector (HPLC-DAD), serial number 8135827, was used. A Thermo Scientific Hypersil BDS C18 column (Waltham, MA, USA), 250 mm long, 4.6 mm in diameter, and with a particle size of 5 µm, was employed. A Sigma-Aldrich cinnabaric acid standard with a purity ≥ 98% was used for quantification. The HPLC-DAD analysis was performed with the column temperature maintained at 25 °C. The mobile phases were acetonitrile (phase A) and water with 0.1% phosphoric acid (phase B). The mobile phase flow rate was 1.0 mL/min, and chromatographic separation was performed using a gradient elution program in which solvent B was maintained at 2% from 0 to 1.5 min, increased to 70% at 4 min and held until 7 min, then returned to 2% at 7.1 min and maintained until the end of the run at 10 min. The total analysis time was 10 min, the injection volume was 20 µL, and the detection wavelength was 430 nm. The quantification range was 10–80 µg/mL, and all injections were performed in triplicate.

### 4.7. Computational Calculations

Density Functional Theory (DFT) [57] was employed to optimize the geometries of cinnabaric acid (CINO) and cinnabarin (CINA). Calculations were carried out in Gaussian [58] using the B3LYP hybrid exchange-correlation functional, chosen for its established balance between computational cost and accuracy in predicting molecular properties; its reliability for electronic, geometric, and energetic properties is well documented [59,60,61], supporting its use for the present systems. Molecular properties were obtained with the Ahlrichs TZVP basis set, which offers a detailed and accurate description of molecular electronic structure [62,63]. Self-consistent field convergence was set to a density-matrix criterion of 10^−14^ a.u., targeting an energy convergence threshold of at least 10^−15^ a.u. (Gaussian keyword: SCF = tight), following Gaussian’s recommended settings to ensure reliable results. Molecular structures were visualized in GaussView 6 (Semichem Inc., Shawnee Mission, KS, USA).

Following geometric optimization of CINO and CINA, periodic DFT calculations on the ZTO-CINO, ZTO-CINA, TO-CINO, and TO-CINA systems were carried out in VASP [64] to elucidate the adsorption mechanisms of both molecules on the ZnTiO_3_ (ZTO) and TiO_2_ (TO) surfaces. The Perdew–Burke–Ernzerhof functional within the generalized gradient approximation (GGA-PBE) [65] was used, with electron–ion interactions described by the projector augmented wave (PAW) method [66]. Kohn–Sham equations [67] were solved self-consistently to an energy variation below 10^−5^ eV between cycles, with a 500 eV plane-wave cutoff. Adsorption of CINA and CINO on the ZTO and TO surfaces was modeled using the previously optimized unit cells: hexagonal ZnTiO_3_ (a = 5.15 Å, b = 5.15 Å, c = 13.94 Å; α = β = 90°, γ = 120°) and tetragonal anatase TiO_2_ (a = 3.82 Å, b = 3.82 Å, c = 9.70 Å; α = β = γ = 90°) [42]. Brillouin-zone sampling employed Monkhorst–Pack k-point meshes [68] of 3 × 2 × 1 for ZTO and 1 × 3 × 1 for TO, with all atomic positions relaxed until residual forces fell below 0.001 eV/Å. Total-energy convergence was improved via Gaussian smearing of band occupations (σ = 0.10 eV). Bandgap values for ZnTiO_3_ (3.17 eV) and TiO_2_ (3.21 eV), together with their conduction- and valence-band edges (ZnTiO_3_: E_CB_ = −0.22, E_VB_ = +2.84 eV; TiO_2_: E_CB_ = −2.06, E_VB_ = +1.06 eV), were taken from our previous GGA+U study [42,48]. All calculations were non-spin-polarized, and molecular models were built and visualized in BioVia Material Studio, version 5.5 (BioVia, San Diego, CA, USA).

Adsorption of CINA and CINO was studied on the stable (101) surface [69,70,71], obtained by cleaving the bulk ZTO and TO crystals. The corresponding adsorption energy (ΔE_ads_) on each oxide surface was obtained from the expression below [72]:

(6) $$∆E_{ads}=E_{adsorbate/adsorbent}-E_{adsorbent}-E_{adsorbate}$$

where E_adsorbate_ is the energy of the supersystem formed by the adsorbed molecule on the surface (eV), E_adsorbent_ is the energy of the clean oxide (eV), and E_adsorbate_ is the energy of the isolated molecule in vacuum (eV).

## 5. Conclusions

This study demonstrates, for the first time, that the phenoxazine pigments cinnabarin and cinnabaric acid, extracted from *Pycnoporus sanguineus*, function as effective dye sensitizers for dye-sensitized solar cells (DSSCs), and that their combination with a ZnTiO_3_/TiO_2_ heterojunction photoanode produces a consistent and mechanistically explainable performance improvement compared to TiO_2_ alone.

Spectroscopic characterization (HPLC-DAD, FTIR) confirmed that cinnabarin and cinnabaric acid are the dominant chromophores of the extract, with a well-defined phenoxazine core and carboxyl/hydroxyl anchoring chemistry. X-ray diffraction (XRD) confirmed the coexistence of hexagonal ZnTiO_3_ and TiO_2_ anatase in the heterojunction photoanode, unlike the single anatase phase present in TO. The photovoltaic data showed a statistically validated improvement in all four device configurations (TO < PSE/TO < ZTO/TO < PSE/ZTO/TO), with efficiency increasing from 0.06% (TO) to 0.38% (PSE/ZTO/TO), and all intermediate descriptors (V_oc_, J_sc_, FF) following the same order (Table 1). The V_oc_ gain of the ZnTiO_3_/TiO_2_ heterojunction persisted even in the comparison of dye-free TO versus ZTO/TO, indicating that the heterojunction’s contribution to performance is electronic and not related to increased light absorption or dye loading.

Band-alignment calculations (Butler–Ginley model) placed the external conduction band (E_CB_) of both dyes between the conduction bands of ZnTiO_3_ and TiO_2_ (Table 2), identifying a thermodynamically favorable, downward injection pathway towards ZnTiO_3_ and a blocked, upward pathway towards TiO_2_ (Figure 13). Because the reverse transition from ZnTiO_3_ to TiO_2_ is energetically less favorable, TiO_2_ acts as a one-way energy gate, confining the photoinjected electrons within the ZnTiO_3_ and suppressing recombination by backtransfer. This directly explains the experimentally observed dye-independent V_oc_ gain.

Finally, DFT adsorption modeling (Table 3) showed that both dyes anchor to either oxide surface predominantly in a vertical orientation, stabilized by an additional Ti–O dative bond beyond the hydrogen-bonding network present in the horizontal configuration. Within this shared anchoring geometry, cinnabaric acid and cinnabarin exhibit differentiated surface selectivity toward ZnTiO_3_ and TiO_2_, respectively. This is consistent with the electronic modulation of the shared amino/carboxyl anchoring end by the non-anchoring distal substituent of each molecule and correlates with their respective dipole moments. This places cinnabaric acid in direct contact with the energetically favorable ZnTiO_3_ injection channel, while cinnabarin’s contribution likely occurs via interfacial transfer or ultrafast injection kinetics, despite its less favorable local band alignment.

In conclusion, these results support the engineering of ZnTiO_3_/TiO_2_ heterostructures, rather than dye optimization alone, as the most important means of improving the performance of DSSCs sensitized with fungal pigments. As an initial prospective investigation, this study evaluated four candidate photoanode/sensitizer architectures (TO, PSE/TO, ZTO/TO, and PSE/ZTO/TO), identifying PSE/ZTO/TO as the best-performing configuration. This architecture therefore provides a rational platform for subsequent studies aimed at further improving device performance. In particular, future work will evaluate the incorporation of rare-earth elements as a strategy to reduce interfacial recombination, together with systematic assessment of the operational stability of the resulting devices under continuous illumination.

## Figures and Tables

**Table 1:** Effect of the different treatments on the average values obtained in this study and their statistical differences.

System Evaluated	TO	PSE/TO	ZTO/TO	PSE/ZTO/TO
Open-Circuit Voltage, V_oc_ (V) (±SDS)	0.42 (±0.010) ^a^	0.45 (±0.020) ^b^	0.47 (±0.010) ^b^	0.5 (±0.015) ^c^
Short-Circuit Current Density, J_sc_ (mA/cm^2^)	0.28 (±0.010) ^a^	0.61 (±0.027) ^b^	0.74 (±0.015) ^c^	1.14 (±0.046) ^d^
Fill Factor, FF	0.48 (±0.011) ^a^	0.55 (±0.024) ^b^	0.55 (±0.013) ^b^	0.67 (±0.027) ^c^
Voltage at Maximum Power Point, V_mp_ (V)	0.29 (±0.007) ^a^	0.33 (±0.015) ^b^	0.35 (±0.007) ^b^	0.40 (±0.016) ^c^
Current Density at Maximum Power Point, J_mp_ (mA/cm^2^)	0.19 (±0.005) ^a^	0.46 (±0.020) ^b^	0.56 (±0.012) ^c^	0.96 (±0.038) ^d^
Efficiency, η (%)	0.06 (±0.001) ^a^	0.15 (±0.008) ^b^	0.19 (±0.005) ^c^	0.38 (±0.015) ^d^

NOTE: Values represent the arithmetic mean of five replicates (*n* = 5) plus the standard deviation. Different letters (a, b, c, d) in the same column indicate statistically significant differences according to Tukey and Duncan post hoc tests (α = 0.05) after an analysis of variance (ANOVA, *p* < 0.001).

**Table 2:** Gaussian calculation summary.

Specifications	Results	
File Name	Cinnabarinic Acid	Cinnabarin
Electronic Energy (eV)	−27,905.24	−29,920.50
Dipole Moment (Debye)	2.50	1.87
Polarizability (a.u.)	228.72	231.71
E (Thermal) (kcal/mol)	152.27	140.80
Heat Capacity (Cv) (cal/mol-ke)	66.69	68.28
Entropy (S) (cal/mol-ke)	130.67	136.16
Homo Energy (eV)	−6.42	−6.44
Lumo Energy (eV)	−3.33	−3.33
Bandgap (eV)	3.09	3.11
Electronegativity (eV)	4.87	4.89
E_CB_ (eV)	−1.175	−1.165
E_VB_ (eV)	1.915	1.945
Point Group	C1	C1

**Table 3:** Adsorption energies of cinnabaric acid and cinnabarin on the surfaces of ZTO and TO.

Semiconductor	Cinnabarinic Acid		Cinnabarin	
	Vertical	Horizontal	Vertical	Horizontal
ZnTiO_3_	−206.07 kJ/mol	−78.45 kJ/mol	−152.91 kJ/mol	−44.82 kJ/mol
TiO_2_	−201.40 kJ/mol	−134.24 kJ/mol	−251.82 kJ/mol	−229.32 kJ/mol

## Data Availability

The original contributions presented in this study are included in the article/Supplementary Material. Further inquiries can be directed to the corresponding author.

## Supplementary Materials

The following supporting information can be downloaded at: https://www.mdpi.com/article/10.3390/molecules31183310/s1, Figure S1. (a) UV spectrum and (b) Chromatogram of the cinnabaric acid standard, Figure S2. Emission spectrum of the General Lighting Led lamp, Table S1. Bonds and interaction distances between cinnabaric acid and cinnabarin molecules and the semiconductors ZnTiO_3_ (ZTO) and TiO_2_ (TO).

## Abbreviations

The following abbreviations are used in this manuscript:

DSSC	Dye-Sensitized Solar Cell
PCE	Power Conversion Efficiency
DFT	Density Functional Theory
PSE	*Pycnoporus sanguineus* Extract
ZTO	Zinc Titanate
TO	Titanate Oxide (Anatase phase)
CINA	Cinnabarine
CINO	Cinnabarinic Acid
J_sc_	Short-Circuit Current Density
V_oc_	Open-Circuit Voltage
V_mp_	Maximum Power Point
J_mp_	Voltage at the Maximum Power Point
E_VB_	Energy of Valence Band
E_CB_	Energy of Conduction Band
E_g_	Energy of Bandgap
E_C_	Energy of Free Electrons
HOMO	Highest Occupied Molecular Orbital
LUMO	Lowest Unoccupied Molecular Orbital
