# Supplementary material for: Phenoxazine-Type Pigments from Pycnoporus sanguineus as ZnTiO3/TiO2 Sensitizers for DSSCs
Source: Molecules. 2026 Sep 18;31(18):3310. doi: 10.3390/molecules31183310 (PMC13609877; doi:10.3390/molecules31183310)
Supplement: Supplementary file 1 [file molecules-31-03310-s001.zip › molecules-4554771-supplementary.pdf]

Supplementary Materials

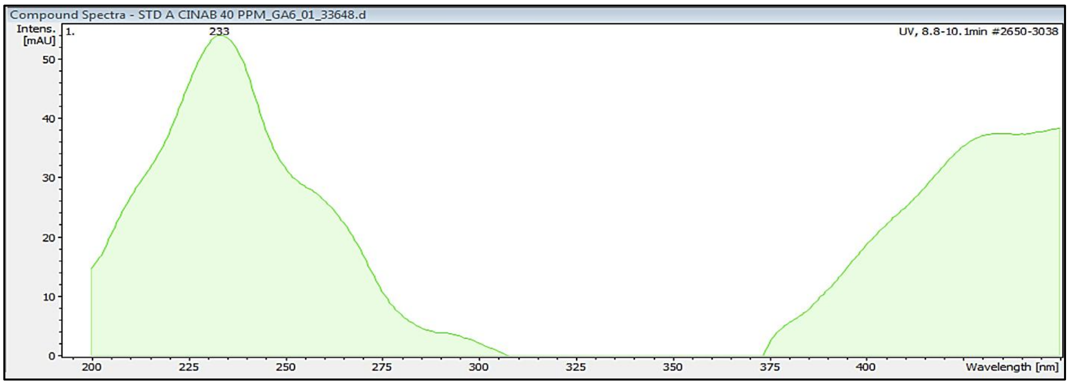

(a)

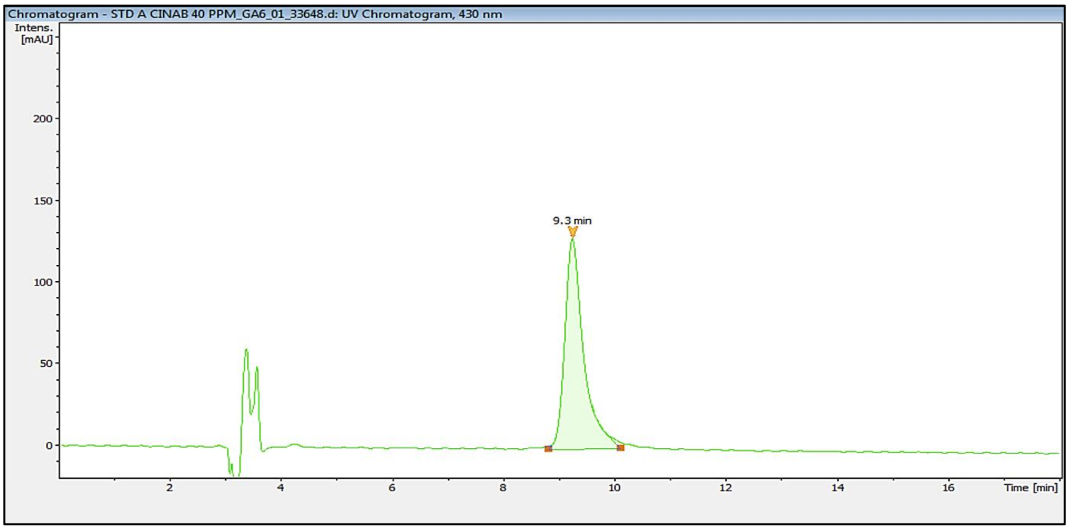

(b)

Figure S1. (a) UV spectrum and (b) Chromatogram of the cinnabaric acid standard

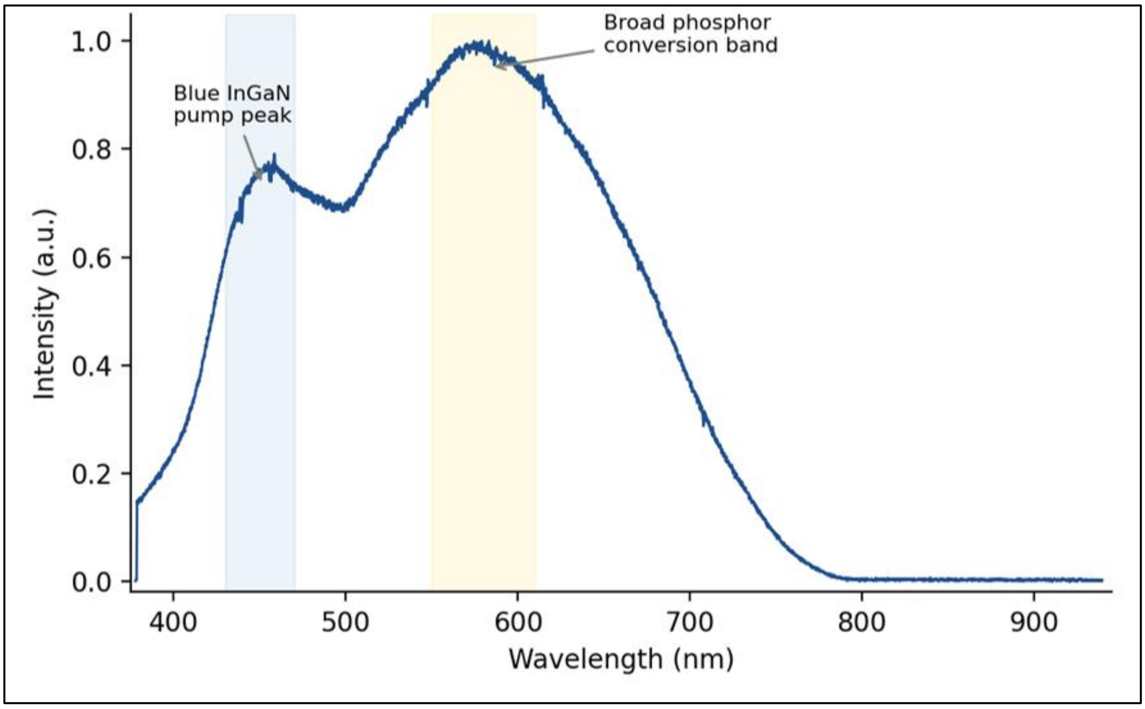

Figure S2. Emission spectrum of the General Lighting Led lamp

Table S1. Bonds and interaction distances between cinnabaric acid and cinnabarin molecules and the semiconductors $ZnTiO_3$ and TiO_2

Sistem	$ZnTiO_3$ (ZTO)			TiO_2 (TO)		
	**Bonds**	**Distances (Å)**	**Type**	**Bonds**	**Distances (Å)**	**Type**
Cinnabarinic Acid - H	H7-O6	1.67	H bond	H7-O66	1.68	H bond
	H8-O4	1.74	H bond	H8-O59	1.63	H bond
Cinnabarinic Acid - V	H8O6	1.56	H bond	H8-O66	1.90	H bond
	H5-O3	2.03	H bond	H5-O55	1.94	H bond
	O113-Ti17	2.04	Covalent bond	O341-Ti57	2.12	Covalent bond
Cinnabarin - H	H9-O6	1.75	H bond	H9-O66	1.75	H bond
	H10-O4	1.74	H bond	H10-O59	1.66	H bond
Cinnabarin - V	H7-O3	2.05	H bond	H10-O66	1.94	H bond
	H10-O6	1.58	H bond	H7-O55	1.93	H bond
	O113-Ti17	2.03	Covalent bond	O341-Ti57	2.13	Covalent bond
